# CD147 a direct target of miR-146a supports energy metabolism and promotes tumor growth in ALK+ ALCL

**DOI:** 10.1038/s41375-022-01617-x

**Published:** 2022-06-08

**Authors:** Ivonne-Aidee Montes-Mojarro, Julia Steinhilber, Christoph M. Griessinger, Achim Rau, Ann-Kathrin Gersmann, Ursula Kohlhofer, Petra Fallier-Becker, Huan-Chang Liang, Ute Hofmann, Mathias Haag, Wolfram Klapper, Elke Schaeffeler, Bernd J. Pichler, Matthias Schwab, Falko Fend, Irina Bonzheim, Leticia Quintanilla-Martinez

**Affiliations:** 1 Institute of Pathology and Neuropathology and Comprehensive Cancer Center Tübingen, Eberhard-Karls-University, Tübingen, Germany; 2 Werner Siemens Imaging Center, Department of Preclinical Imaging and Radiopharmacy, Eberhard-Karls-University, Tübingen, Germany; 3 Department of Pathology, Unit of Experimental and Laboratory Animal Pathology, Medical University of Vienna, Vienna, Austria; 4 Dr. Margarete Fischer-Bosch Institute of Clinical Pharmacology, Stuttgart, Germany; 5 Eberhard-Karls-University, Tübingen, Germany; 6 Hematopathology Section, University Hospital Schleswig-Holstein Campus Kiel/Christian-Albrechts University Kiel, Kiel, Germany; 7 Cluster of Excellence iFIT (EXC2180) “Image-guided and Functionally Instructed Tumor Therapies”, Eberhard-Karls-University, Tübingen, Germany; 8 German Cancer Consortium (DKTK) and German Cancer Research Center (DKFZ), Partner Site Tübingen, Tübingen, Germany; 9 Departments of Clinical Pharmacology, Pharmacy and Biochemistry, Eberhard-Karls-University, Tübingen, Germany

**Keywords:** T-cell lymphoma, Oncogenesis

## Abstract

We recently reported that miR-146a is differentially expressed in ALK+ and ALK− anaplastic large cell lymphoma (ALCL). In this study, the downstream targets of miR-146a in ALK+ ALCL were investigated by transcriptome analysis, identifying *CD147* as potential target gene. Because CD147 is differentially expressed in ALK+ ALCL versus ALK− ALCL and normal T cells, this gene emerged as a strong candidate for the pathogenesis of this tumor. Here we demonstrate that CD147 is a direct target of miR-146 and contributes to the survival and proliferation of ALK+ ALCL cells in vitro and to the engraftment and tumor growth in vivo in an ALK+ ALCL-xenotransplant mouse model. CD147 knockdown in ALK+ ALCL cells resulted in loss of monocarboxylate transporter 1 (MCT1) expression, reduced glucose consumption and tumor growth retardation, as demonstrated by [^18^F]FDG-PET/MRI analysis. Investigation of metabolism in vitro and in vivo supported these findings, revealing reduced aerobic glycolysis and increased basal respiration in CD147 knockdown. In conclusion, our findings indicate that CD147 is of vital importance for ALK+ ALCL to maintain the high energy demand of rapid cell proliferation, promoting lactate export, and tumor growth. Furthermore, CD147 has the potential to serve as a novel therapeutic target in ALK+ ALCL, and warrants further investigation.

## Introduction

Anaplastic large cell lymphoma (ALCL) is a T-cell non-Hodgkin lymphoma with anaplastic morphology and strong CD30 expression [1]. Two distinct disease entities are recognized based on the expression or lack of the anaplastic lymphoma kinase (ALK). ALK+ ALCL frequently carries the chromosomal translocation t(2;5)(p23;q35) involving the anaplastic lymphoma kinase (*ALK*) and the nucleophosmin (*NPM*) gene, leading to the expression and constitutive activation of chimeric ALK fusion protein. ALK fusion proteins activate several key signaling pathways involved in transformation, cell proliferation and survival, including STAT3, AKT/mTOR, RAS/MAPK, PLCγ and PI3K [2]. A central target gene of the JAK/STAT signaling pathway is the transcription factor C/EBPβ, which is overexpressed in ALK+ ALCL [3–5]. C/EBPβ is also able to control tumorigenesis through regulation of gene expression by miRNA up- and downregulation [6]. Several deregulated miRNA have been identified in different studies, including the downregulated miR-16, miR-21, miR-26a, miR-29, miR-96, miR-101, miR-146a and miR-155; besides the upregulated miR-135b and miR-17-92 cluster, suggesting that these miRNAs might contribute to ALK-mediated oncogenesis and/or tumor biology [7–11]. So far only few target genes of deregulated miRNAs have been revealed in ALK+ ALCL. *ZNF652*, *BACH1*, *RBAK*, *E2F2* and *TP53INP1* were detected as target genes of miR-155 [12], whereas *MCL-1, INOS and ALK* are target genes of miR-29a, miR-26a and miR-96, respectively [7, 10, 13]. *FOXO1*, *STAT6* and *GATA3* were identified as target genes of the highly expressed miR-135b [9].

The intrinsic low expression of miR-146a previously reported in ALK+ ALCL in comparison to ALK− ALCL, normal T cells and lymph nodes [6, 8], suggested that this miRNA might have an impact in the tumorigenesis of ALK+ ALCL. Importantly, miR-146a has been found to function as a potent tumor suppressor gene, is associated with the T-helper 1-(Th1)-phenotype and is involved in the development of lymphoid neoplasms [14]. Furthermore, in hepatocellular carcinoma (HCC) and non-small cell lung carcinoma (NSCLC), the hypermethylation of the promoter of miR-146a is associated with increased cell proliferation, cell survival, invasion, migration and metastasis. Hence, the use of miR-146a appears to be effective in the treatment of NSCLC patients [15, 16].

Therefore, the aim of this study was to identify miR-146a target genes by transcriptome analysis using next generation sequencing (NGS) in ALK+ ALCL and further analyze relevant candidates.

## Materials and methods

### Cell culture and patient samples

Five ALK+ ALCL cell lines (SUDHL-1, KiJK, Karpas 299, SUP-M2 and SR-786), three ALK− ALCL cell lines (Mac-1, Mac2a and FE-PD), HEK293T and HeLa cells were cultured as previously described (Supplementary Methods) [3, 17]. Formalin-fixed paraffin embedded (FFPE) primary tumor samples of 81 ALK+ and 14 ALK− ALCLs patients were collected from the archives of the Institutes of Pathology from the University of Tübingen and the University of Kiel, Germany. Ethics approval for the study (620/2011BO2) was obtained from the University of Tübingen.

### RNA isolation

Total RNA and miRNA were isolated from cell lines using the RNeasy Mini Kit and miRNeasy Mini Kit, respectively (Qiagen, Hilden, Germany) (Supplementary Methods).

### Overexpression of miR-146a

Overexpression of miRIDIAN miRNA Mimic 146a, Mimic Housekeeping Positive Control #2 (GAPD) and miRIDIAN microRNA Mimic Negative Control #1 (GE Healthcare, Buckinghamshire, United Kingdom) was performed in SUDHL-1 or Karpas 299 (Supplementary Methods).

### Transcriptome analysis

Transcriptome analysis using NGS was performed by CeGaT (Tübingen, Germany) (Supplementary Methods).

### Real-time quantitative RT-PCR

cDNA synthesis from RNA and real-time quantitative RT-PCR analysis (RT-qPCR) to quantify the mRNA level of CD147 was performed using Universal Probe Library (UPL) assays (Roche Applied Science, Penzberg, Germany). Mature miRNA quantification, cDNA synthesis and RT-qPCR analysis were performed as previously described (Supplementary Methods) [18].

### Western blot analysis

Lysis of cells and Western blotting were performed as described elsewhere [4, 19]. The antibodies used are listed in the Supplementary Information.

### Cloning of CD147-shRNA constructs, Virus Production and Viral Infections

Oligonucleotides containing CD147-shRNA sequences were cloned into pSUPER and pFUGW (pF-CD147), as previously described (Supplementary Methods) [3]. Production of virus containing lentiviral vector pFUGW was performed as recently described [20]. Transduction efficiency was determined as previously specified [5, 17, 19].

### CD147 CRISPR/Cas-9 system knockout

Individual lentiviral CRISPR plasmids targeting a single genomic locus - CD147 - were designed and constructed according the lentiCRISPRv2 #52961, (Addgene Watertown, Massachusetts, USA), as previously described [21]. Pathogenic mutations leading to CD147 protein damage were investigated with targeted NGS as previously reported (Supplementary Methods).

### Luciferase reporter assay

The regions of the CD147 and SRPRB 3´-UTRs, including the miR-146a binding site predicted by the miRanda tool were amplified from human genomic DNA using PCR [22]. For luciferase reporter assays, HEK293T and HeLa cells were transfected (Supplementary Methods).

### Cell proliferation and viability assay (MTS assay)

Cell viability and growth retardation was determined by the MTS cell proliferation assay (AQueous CellTiter 96, Promega) [3, 23]. Apoptosis analysis was done by annexin V (AnnexinV-APC, Invitrogen) and propidium iodide (PI) (Sigma-Aldrich) stainings according to manufacturer protocols, followed by flow cytometry (FACS Calibur, BD, Franklin Lakes, NJ, USA).

### Crizotinib treatment

ALK+ ALCL cells were treated with increasing concentrations of Crizotinib (25, 50 and 100 nM). Protein and miRNA were isolated after 72 h (Supplementary Methods).

### Experimental mice

All animal experiments were approved by the Regierungspräsidium Tübingen and performed according to animal use and care protocols of the German Animal Protection Law (Supplementary Methods).

### Sequential PET/MRI

For the PET-measurements, the tracer [^18^F]FDG was used, which was synthesized in a FDG MicroLab module (GE Healthcare, Münster, Germany) as described previously [24]. Sequential [^18^F]FDG-PET/MRI scans were performed 3 and 4 weeks after tumor cell implantation (Supplementary Information).

### Immunohistochemistry

Explanted xenograft and primary ALCL tumors were stained with haematoxylin and eosin (H&E). Immunohistochemistry was performed on an automated immunostainer (Ventana Medical Systems, Inc.) according to the company’s protocols for open procedures (Supplementary Information).

### Transmission electron microscopy (TEM)

FFPE materials were used for the analysis (Supplementary Information).

### Non-targeted metabolomics

Metabolomic profiling was performed by LC-QTOF-MS analysis as described [25] (Supplementary Information). Data were preprocessed by targeted feature extraction of annotated metabolite species [25]. Peak areas were log2 transformed and normalized by median normalization prior the assessment of fold changes between CD147 knockdown (KD) tumors and controls.

### Targeted metabolomics

Pyruvate, aconitate, fumarate, α-ketoglutarate, malate and citrate were quantified by GC-MS analysis and lactate by LC-MS-MS as described previously (Supplementary Information) [26–29].

### XF cell mito stress test using seahorse XFe96 analyzer

SUDHL-1 and KiJK cells (control and CD147-KD) were grown in suspension after viral infection and incubated for at least 72 h. Oxygen consumption rates (OCR) were measured using Agilent Seahorse XFe96 Analyzer (Seahorse Bioscience; Agilent Technologies, Inc., Santa Clara, CA, USA) and the Seahorse XF Cell Mito Stress Test Kit (Agilent) [30], according to the manufacturer’s protocol (Supplementary Information).

### Measurement of mitochondrial membrane potential (ΔΨm) using FACS analysis

SUDHL-1 cells, SUDHL-1 cells with CD147-KO and SUDHL-1 cells under stress conditions (6 days starvation) were stained with Mito Tracker Green (MG) and Mito Tracker Deep Red (MDR) (ThermoFisher Scientific). FACS analysis was performed and data were analyzed using FlowJo V9.9.6 (Supplementary Information) [31].

### Statistical analysis

Specific details concerning statistical tests for individual experiments are noted in the figure legends and in Supplementary Information. *P* values <0.05 were considered significant.

## Results

### Identification of miR-146a target genes by transcriptome analysis using NGS

**Fig. 1:**
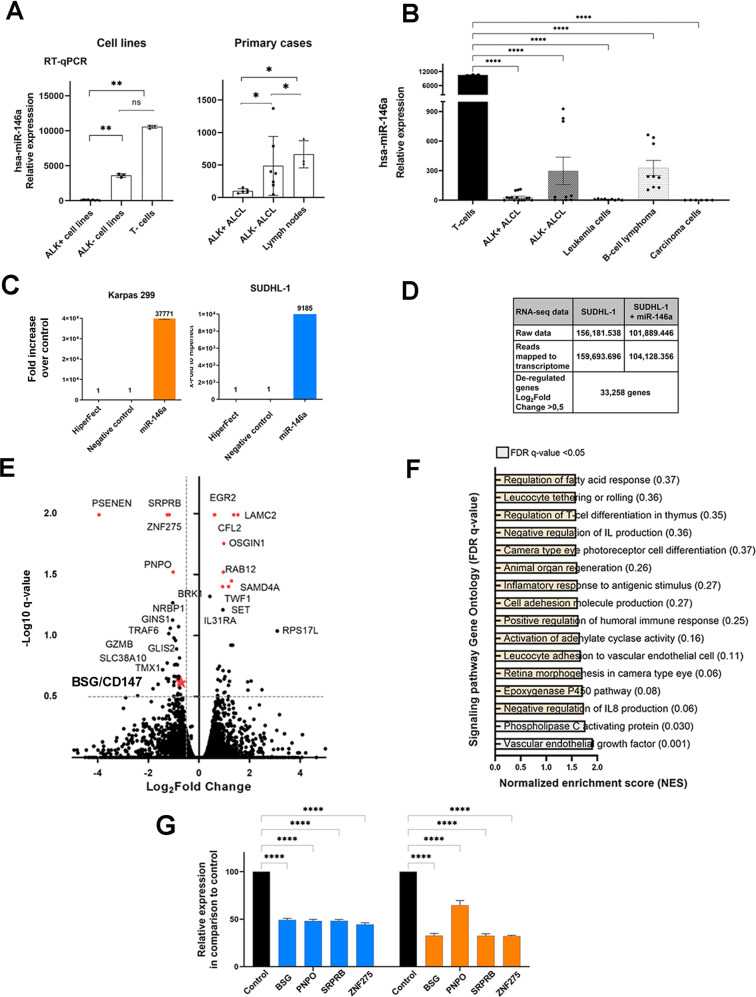
miR-146a target genes. **A** RT-qPCR results of miR-146a expression in primary ALK+ ALCL cases (ALK+ five cases, ALK− seven cases and four reactive lymph nodes) and cell lines. For cell line analysis ALK+ ALCL cell lines (SUDHL-1, KiJK, Karpas 299) were compared to the ALK− ALCL cell line (Mac-1) and CD3+ T cells from 3 healthy donors. Every point represents the average of three independent measurements. For RT qPCR quantifications values were normalized to miR-106b and analyzed using the 2^−ΔΔCp^ method [6]. **B** Comparison of miR146a expression levels in ALK+ ALCL cells (SUDHL-1, Karpas 299, KiJK, SR786, SUP-M2), ALK− ALCL cells (FE-PD, Mac-1 and Mac2a), CD3+ T cells from 3 healthy donors, leukemia cells (Jurkat and HL-60), B-cell lymphoma cells (SUDHL-4, jeko-1 Rec-1 and and KMS-12) and carcinoma cell lines (HeLa, HEK293-T). Every point represents the average from three independent measurements. For RT qPCR quantifications values were normalized to miR-106b and analyzed using the 2^−ΔΔCp^ method. **C** RT-qPCR results of relative miR-146a expression levels in untransfected, and miR-146a mimic-transfected SUDHL-1 (blue) and Karpas 299 (orange) cells. For RT-qPCR quantification, values were normalized to miR-106b, and data were analyzed according to the 2^−ΔΔCp^ method. Results are depicted as miRNA levels relative to mean value levels of HiperFect treated cells. **D** Overview of the reads generated in the transcriptome analysis by NGS, and number of deregulated genes comparing control/ miR-146a overexpression. **E** Volcano plot of RNA-seq transcriptome data displaying the differentially expressed genes (FDR-corrected *p* ≤ 0.05). Significantly regulated genes after miR-146a overexpression (>0.5 Log2 Fold change) are depicted with orange dots (four downregulated genes left side). Coordinates of CD147 is marked with an orange star. **F** Bar graphs represent Gene Ontology pathways enriched in the Gene Set Enrichment Analysis (GSEA) of the differentially expressed genes ranking list. Signaling pathway with FDR q-value <0.05 are highlighted in gray. **G** RT-qPCR analysis of miR-146a regulated target genes. Relative expression levels of the four selected candidate genes (*ZNF275*, *SRPRB*, *PNPO* and *CD147*) regulated by miR-146a were determined by RT-qPCR analysis in miR-146a transfected or untransfected SUDHL-1 (blue) and Karpas 299 (orange) cells. Relative mRNA downregulation of the candidate genes was identified using RT-qPCR quantification to ACTB and data were analyzed according to the 2^−ΔΔCp^ method. Each plot represents the average from biological triplicates. For statistical analysis unpaired *t*-test (target *vs* control) was performed. Results are depicted as mRNA levels relative to mean value levels of HiperFect treated SUDHL-1 and Karpas 299 cells (control).

**Fig. 2:**
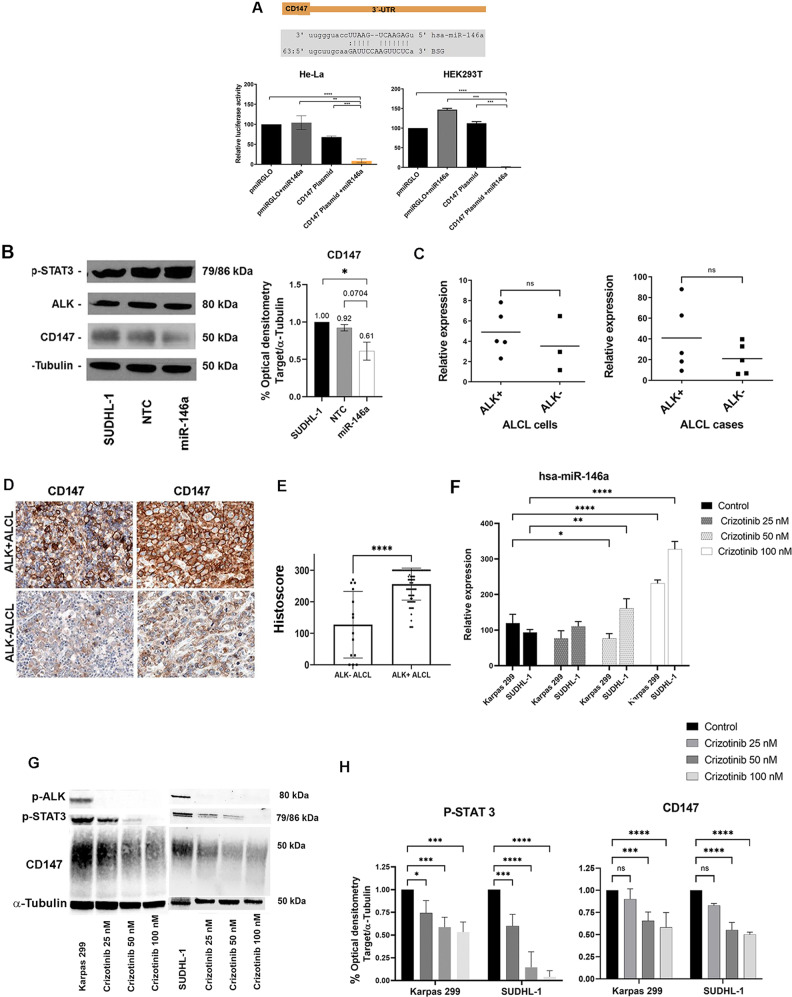
CD147 is a target gene of mi-146a and is differentially expressed in ALK+ and ALK− ALCL. **A** Luciferase activity using the pmirGLO Vector containing CD147 region targeted by miR-146a. Upper panel illustrates miR-146a binding site within the 3´-UTR of CD147 according to miRanda prediction tool (www.microrna.org/). Lower panel displays the dual luciferase reporter assay performed in HeLa and HEK293-T cells co-transfected using the pmiRGLO vector with and without CD147-3´-UTR construct. Relative luciferase activity was measured in triplicates (firefly LUC / renilla LUC) after 40 h of transfection. Columns represent mean luciferase activity in HEK293-T and HeLa cells transfected. For statistical Unpaired *t*-test was used, **p < 0.01, ***p < 0.001, ****p < 0.0001. **B** Western blot analysis of ALK, p**-**STAT-3 and the miR-146a target protein CD147 in ALK+ ALCL SUDHL-1 cells with miR-146a transfection in comparison to controls (SUDHL-1 untransfected and transfected with non-targeted mi-RNA). Western blot analysis demonstrates reduced CD147 protein and unaffected expression of ALK and p-STAT3 after miR-146a overexpression. Each lane contained 15 µg protein extract. α-Tubulin was used as loading control. CD147 quantification was obtained from independent biological triplicate samples and measured by optical densitometry. Values were normalized to α-tubulin and represented in the bar graph. For statistical analysis unpaired *t*-test was used, *p < 0.05. **C** mRNA levels of CD147 were investigated in five ALK+ (SUDHL-1, KiJK, Karpas 299, SUP-M2 and SR-786) and three ALK− (Mac-1, Mac2a and FE-PD) ALCL cell lines and in five primary ALK+ and ALK− ALCL cases using RT-qPCR. RT-qPCR quantification values were normalized to ACTB. Results are depicted as plots representing the ratio of ACTB/ target gene Cp-values, each point represents the average of three independent measurements. For statistical analysis a Wilcoxon rank-sum test was used (*p* = 0.28 and *p* = 0.21, respectively). **D**. ALK+ ALCL cases display strong and homogenous membranous CD147 expression. In contrast, ALK− ALCL cases reveal a weaker expression of CD147 (Immunohistochemistry; original magnification 400×). **E** Immunohistochemistry quantification of CD147 staining in primary cases: 81 cases ALK+ ALCL and 14 ALK− ALCL cases. Every point represents a case. For statistical analysis a Wilcoxon rank-sum test was used, ****p < 0.0001. **F** RT-qPCR analysis of relative miR-146a expression levels in corresponding ALCL cells with increasing doses of NPM-ALK inactivation. Each bar represents the average of biological triplicates. For statistical analysis unpaired *t*-test was used, **p < 0.01, ***p < 0.001, ****p < 0.0001. **G** Western blot analysis of p-ALK, p-STAT-3 and CD147 in ALK+ ALCL cell lines (SUDHL-1 and Karpas 299) after 48 h treatment with different doses of Crizotinib, as indicated. Untreated cells were loaded as control. Western blot analysis demonstrates a complete absence of ALK activity after Crizotinib treatment and a reduction of CD147 and p-STAT3 protein expression. Each lane contained 25 µg protein extract. α-Tubulin was used as loading control. **H** Quantification of p-STAT3 or CD147 obtained from independent biological triplicate samples, normalized to α-Tubulin and represented in the bar graph. For statistical analysis unpaired *t*-test was used, *p < 0.05, ***p < 0.001, ****p < 0.0001.

In a previous study, we demonstrated that ALK+ ALCL cell lines and ALK+ ALCL primary cases show very low miR-146a expression in comparison to the moderate expression (4 folds higher) in ALK− ALCL cases and the high expression of reactive lymph nodes (7 folds higher) (Fig. 1A) [6]. miR-146a expression levels are high in T cells (≥10 folds higher) in comparison to T- and B-cell lymphomas, leukemia and carcinoma cell lines (Fig. 1B, Supplementary Fig. 1). In order to investigate the downstream targets of the tumor suppressor miR-146a, ALK+ ALCL cell lines were transfected with miR-146a mimic and analyzed using RNA-seq analysis. ALK+ ALCL cell lines SUDHL-1 and Karpas 299 showed strong overexpression of miR-146a (Fig. 1C). Efficiency of the transfection was confirmed by parallel GAPDH downregulation by the miR-positive control (Supplementary Fig. 2). SUDHL-1 cells transfected with miR-146a or untransfected were analyzed by RNA-seq analysis using NGS. For both samples more than 100 million reads were generated, which were mapped to the human genome. RNA seq data showed 113 genes with statistically differential expression (-log10 q-value > 0.5, log2Fold Change > 0.5) (Fig. 1D, Supplementary Table 1). GSEA of a ranked list of differentially expressed genes revealed higher enrichment score (NES) of gene ontology pathways related to vascular endothelial growth factor and phospholipase C activating protein (FDR q-value < 0.5), supporting the angiogenic role of miR-146a (Supplementary Fig. 3, Fig. 1F) [32]. Eight candidate genes were selected for further validation including the four strongest downregulated genes (*PSENEN, SRPRB, ZNF275, PNPO)*, and four genes with high expression and known oncogenic functions related to ALK+ ALCL (*BSG/CD147, CASP2, ADAM17, PIK3AP)* (Supplementary Table 1, Supplementary Fig. 4). Validation of these eight genes by RT-qPCR confirmed a strong downregulation of four genes; *ZNF275, SRPRB, PNPO* and *CD147* (BSG/basigin, EMMPRIN) to 44–54% in SUDHL-1 and 31–67% in Karpas 299 cells after miR-146a overexpression compared to untransfected control cells (Fig. 1G). A potential direct regulation was investigated for two genes: *CD147* and *SRPRB*. Relative luciferase activity was strongly reduced for *CD147* and *SRPRB* in both cell lines after transfection of miR-146a indicating that both genes are direct targets of miR-146a (Fig. 2A, Supplementary Fig. 5). Because we recently reported that CD147 is differentially expressed in ALK+ ALCL [33], we concentrated further on the analysis of this target gene. Western blot analysis showed downregulation of CD147 after miR-146a overexpression in ALK+ ALCL SUDHL-1 cells compared to controls (Fig. 2B). These results confirmed further the regulation of CD147 protein by miR-146a.

To see whether *CD147* mRNA expression negatively correlated with miR-146a levels, *CD147* mRNA was analyzed in five ALK+ ALCL cell lines (SUDHL-1, KiJK, Karpas 299, SUP-M2 and SR-786) and in three ALK− ALCL cell lines (FE-PD, Mac-1 and Mac2a) using RT-qPCR. ALK+ ALCL cell lines showed stronger expression of *CD147* mRNA when compared to ALK− ALCL cell lines (Fig. 2C). Additionally, *CD147* mRNA levels were investigated in five ALK+ and five ALK− ALCL primary patient samples. ALK+ cases showed higher mRNA levels than ALK− ALCL cases, (*p* = 0.07) (Fig. 2C). To corroborate the differential expression of CD147 protein in primary cases, 95 ALCL cases were investigated (ALK+, 81 cases and ALK− 14 cases). ALK+ ALCL cases revealed a moderate to strong positive membranous staining in the majority of tumor cells, as opposed to the weak or absent staining observed in ALK− ALCL cases (histoscore 264.64+/− 40 *vs* 127.14+/− 105, *p* = 0.0001, Fig. 2D, E). To investigate whether the expression of CD147 and miR-146a were ALK-dependent, two ALK+ ALCL cell lines where treated with increasing concentrations of Crizotinib, which led to inhibition of P-ALK and P-STAT3 with subsequent increase in miR146a (Fig. 2F), and decrease in CD147 (Fig. 2G, H). These results indicate that ALK regulates the expression of miR-146a and CD147.

### CD147 contributes to the survival and proliferation of ALK+ ALCL cells in vitro

**Fig. 3:**
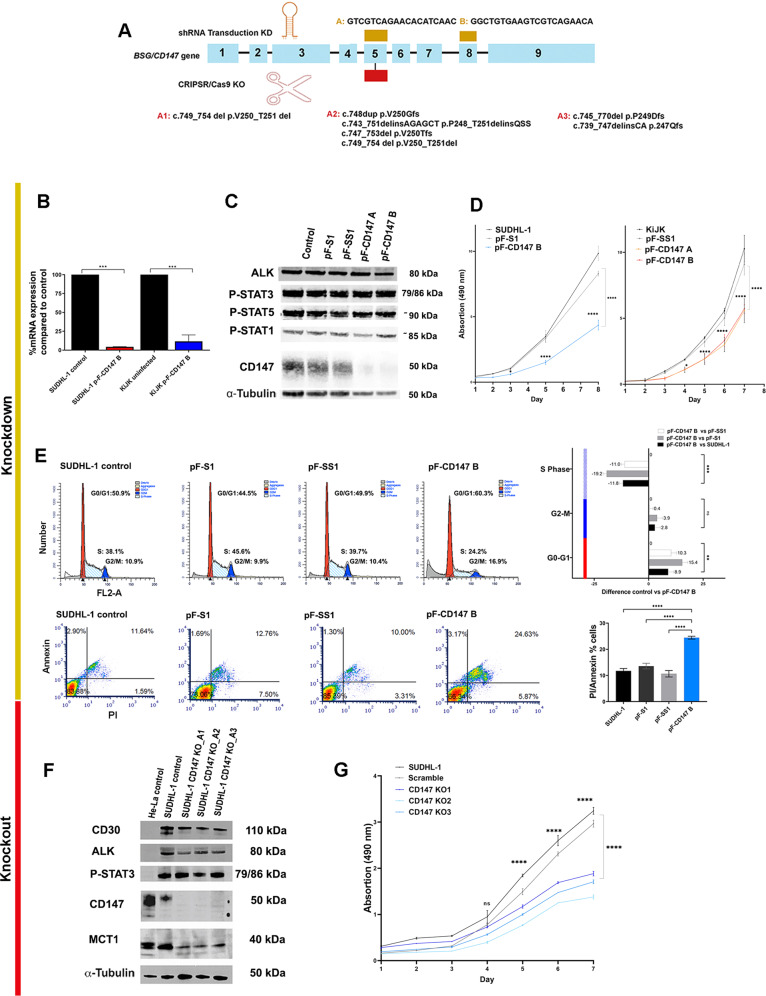
CD147 contributes to the survival and proliferation of ALK+ ALCL cells in vitro. **A** Overview of CD147 silencing using shRNA transduction and CRISPR/Cas 9 editing system. The upper diagram depicts the shRNAs harpins and their target regions (gold rectangles) within the CD147 gene, whereas the lower diagram illustrates the target exon of CD147 and the mutations after CRIPSR/Cas9 editing (red rectangle). **B** RT-qPCR analysis of CD147 mRNA in the transduced SUDHL-1 and KiJK cells three days after second infection. Values were normalized to ACTB and data were analyzed according to the 2^−ΔΔCp^ method. Results are depicted as mRNA amount relative to untreated SUDHL-1 cells. Error bars indicate standard deviation (SD, *n*  =  3). For statistical analysis ANOVA for repetitive measurements was used (*p < 0.05, **p < 0.01 vs. control group). **C** Western blot analysis of CD147, ALK and p-STAT3, p-STAT5 and p-STAT1 in SUDHL-1 cells transduced with CD147 shRNA four days after infection. Blots were performed by triplicates; one representative replicate is depicted. Thirty µg protein were loaded and α-Tubulin was used as loading control. **D** Proliferation curves of the controls and CD147-shRNA infected SUDHL-1 and KiJK cells are depicted up to 8 days after infection. Error bars indicate error of the mean (SEM), (*n*  =  3). Statistical analysis was performed using unpaired *t*-test (for only two group comparison) and repeated measures ANOVA (pFCD147 vs pF, pF-S, and pFSS1) significant differences at different time points and ending point are indicated as **p < 0.001 ****p < 0.0001. Control =  uninfected cells, pF -scrambled = virus containing non-targeted shRNA sequence, pF-CD147 B =  virus containing the CD147 shRNA sequence, CD147-KD B. **E** Cell cycle distribution of the SUDHL-1 controls and CD147-KD cells four days after infection (pF.CD147B vs uninfected, pF-S, and pFSS1). The differences of cell cycle distribution between controls and CD147-KD cells are depicted in graph plots, every plot represents the median and standard deviation (SD). Percentage of annexin/PI staining cells of controls and infected cells are also depicted. Every plot represents the median and SD of the percentage of staining cells of the biological triplicates. Statistical analysis was performed using unpaired *t*-test. Ns = non-significant, *p < 0,05, **p < 0.01, ***p < 0.001. **F** Western blot analysis of MCT1, CD147, P-STAT3, ALK and CD30 in SUDHL-1 after CRISPR/Cas9 editing. Sixty µg of protein were loaded and α-Tubulin was used as loading control. Lysates of HeLa and SUDHL-1 untreated cells served as controls. **G** Proliferation curves of the controls and CD147-KO SUDHL-1 cells are depicted. Error bars indicate SEM (*n * =  3). Statistical analysis was performed using unpaired *t*-test (for only two group comparison) and repeated measures ANOVA (CD147 KO1-3 vs control) significant differences at different time points and ending point are indicated as *p < 0.05, **p < 0.005, ***p < 0.0005. SUDHL-1 control =  uninfected cells, KO 1-2 and 3: three different knockout (KO) clones with different mutations.

To evaluate the effect of CD147 in ALK+ ALCL cells, a double approach was pursued using *CD147* shRNA KD and CD147 knockout (KO) by CRISPR/Cas9 system (Fig. 3A). *CD147* shRNA was selected by testing 5 different shRNA constructs (Supplementary Fig. 6A). *CD147* shRNA “A” and “B” were selected for further analyses and transduced in SUDHL-1 and KiJK (Fig. 3, Supplementary Fig. 6B–E). Flow cytometric analysis showed that CD147-shRNA (pF-CD147) was effectively transduced into the cell lines with infection rates of 97.8% to 99.45% with both shRNAs (Supplementary Fig. 6C). Three days after the second transduction, CD147 was successfully downregulated at mRNA and protein level, as demonstrated by Western blot and RT-qPCR analyses (Fig. 3B, C, Supplementary Fig. 6C, D). RT-qPCR confirmed the *CD147* mRNA downregulation to 8% or 4% in KiJK and SUDHL-1 cells compared to control cells, respectively. Growth curves were generated after seven or eight days of the second infection demonstrating growth retardation of 70% in SUDHL-1 using *CD147* shRNA B or of 59% in SUDHL-1 and 47% in KiJK using *CD147* shRNA A (Fig. 3D, Supplementary Fig. 6E), despite the normal expression of ALK, P-STAT3, P-STAT5 and P-STAT1 (Fig. 3C). Cell cycle analysis demonstrated a decrease in S phase of 11–19.2% in CD147-KD cells in comparison to controls, and an increase in G0/G1 of 8.9–15.4% after four days of infection (Fig. 3E), corroborating a G0/G1 cell cycle arrest. Flow cytometric analysis with annexin V/propidium iodide revealed increased apoptosis when compared to the control cells (11.6–24.6%) (Fig. 3E).

*CD147*-KO after CRISPR/Cas9 editing was confirmed at protein level for three cell clones harboring different *CD147* damaging mutations. Western blot analysis demonstrated complete lack of CD147 protein that correlated with reduced MCT1 expression but normal CD30, ALK and pSTAT3 expression (Fig. 3F). Growth curves of the different SUDHL-1 clones show a growth retardation of 48–68% in comparison to the untreated control after 10 days (Fig. 3G).

### CD147 contributes to tumor growth of ALK+ ALCL cells in vivo

**Fig. 4:**
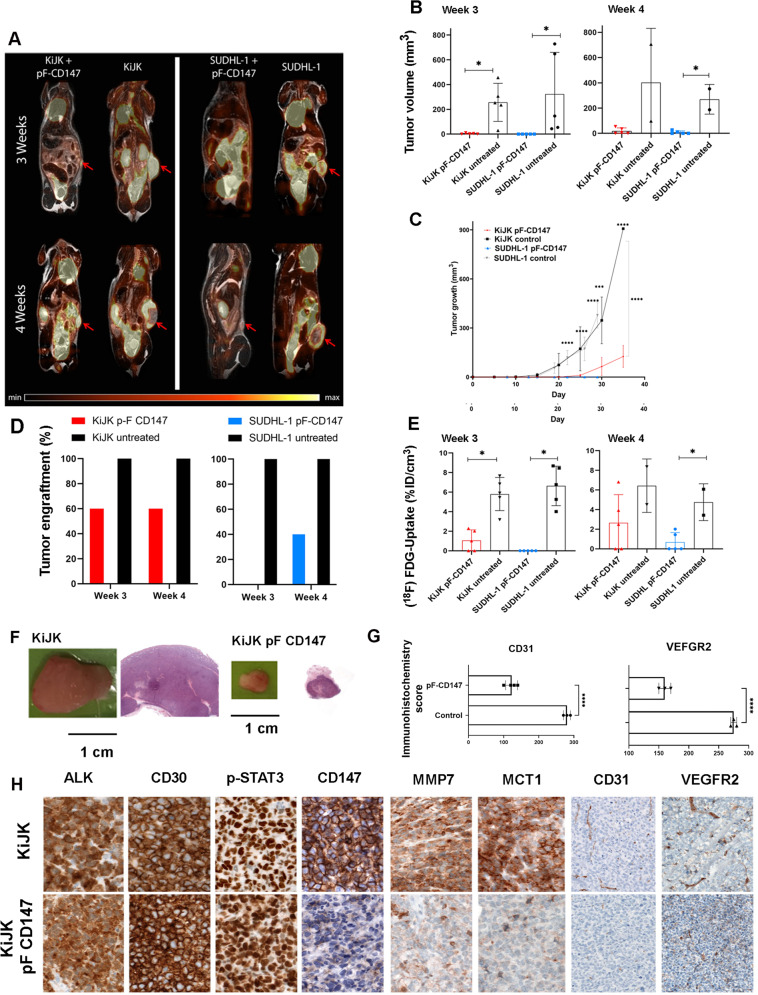
Detection of tumor growth, engraftment and glucose consumption of CD147-KD KiJK- and SUDHL-1 lymphoma cells in mouse xenotransplant models. **A** Exemplary PET/MR-scans of the tumor growth dynamic 3 and 4 weeks after implantation of CD147-KD (pF.CD147) KiJK and SUDHL-1 lymphoma cells (red arrows indicate tumors). **B** MRI-derived tumor volume 3-, and 4-weeks post tumor cell implantation (mean ± SD; CD147-KD: *n* = 5; untreated: *n* = 5, each symbol represents a mouse tumor measurement). CD147-KD precludes growth in KiJK and SUDHL-1 lymphoma cells. Two controls are included at week 4 because 3 of 5 mice died after week 3. For statistical analysis Wilcoxon rank-sum test was used, *p < 0.05. **C** MRI-derived tumor volume growth until day 29 post tumor cell implantation (mean ± SD; CD147-KD: *n* = 5; untreated: *n* = 5, SD is depicted by non-continuous lines). CD147-KD resulted in reduced tumor growth in KiJK and SUDHL-1 cells. Statistical analysis was performed using unpaired *t*-test (for only two group comparison) and repeated measures ANOVA: Significant differences at different time points and ending point are indicated as **P < 0.001 ****P < 0.0001. **D** The engraftment (%) of KiJK- and SUDHL-1-lymphomas was reduced after pF-CD147-treatment (KiJK: CD147-KD *n* = 5, untreated *n* = 5; SUDHL-1: CD147-KD *n* = 5, untreated *n* = 5). **E** [^18^F]FDG-uptake was reduced after CD147-KD in KiJK and SUDHL-cells (mean ± SD; CD147-KD: *n* = 5; untreated: *n* = 5, each symbol represents a mouse tumor measurement). For statistical analysis Wilcoxon rank-sum test was used, *p < 0.05. **F** Macroscopic images of representative tumors derived from the mice described in **A** and Hematoxylin and Eosin (H&E) staining of representative tumors of CD147-KD and untreated KiJK cells (original magnification 12.5×). **G** Graph plots of CD31 and VEFGR2 immunohistochemistry quantification of control tumors (*n* = 3) and tumors with CD147-KD cells (KiJK; *n* = 3). For statistical analysis Unpaired *t*-test ****p < 0.0001). **H** Comparative immunohistochemical analysis of representative tumors of CD147-KD and untreated KiJK cells stained with ALK, CD30, p-STAT3, CD147, MMP7, MCT1, (Immunohistochemistry, original magnification 400×), CD31 and VEGFR2 (Immunohistochemistry, original magnification 200×).

To further investigate the influence of CD147 in survival and tumor growth of ALK+ ALCL cells in vivo, KiJK and SUDHL-1 cells with (*n* = 10; 5 KiJK, 5 SUDHL-1) and without (*n* = 10; 5 KiJK, 5 SUDHL-1) CD147-KD were subcutaneously implanted in 6–8 weeks old female NOD scid gamma immune deficient mice and analyzed by PET/MRI (Fig. 4A–C). After 3 and 4 weeks, KiJK + CD147-KD-tumors engrafted in only 3/5 (60%) animals, as compared to 5/5 (100%) of the animals that received untreated KiJK cells (Fig. 4D), and were 75-fold smaller compared to untreated KiJK-tumors (CD147-KD: 3.42 ± 4.37 mm^3^ vs untreated: 256.4 ± 153.7 mm^3^), (Fig. 4B, F). After 4 weeks most of untreated KiJK-lymphomas reached already the final tumor stage and the animals were sacrificed before the last PET/MRI measurements. Similar results were obtained after implantation of SUDHL-1 + CD147-KD tumor cells, where apparent engraftment was visible after 4 weeks in only 2/5 animals (40%), (Fig. 4A–C) compared to 100% (5/5) in untreated SUDHL-1 cells (CD147-KD: 9.4 ± 12.06 mm^3^; untreated: 324.02 ± 336.117.2 mm^3^) (Fig. 4B). These results clearly demonstrate that CD147 is necessary for engraftment and growth of ALK+ ALCL cells.

To corroborate the lack of CD147 expression in CD147-KD cells, immunohistochemical analysis was performed in the murine tumors (Fig. 4H). Tumors with CD147-KD showed complete lack of CD147 expression, as compared to the untreated cells. Lack of CD147 expression correlated with lack of MCT1 expression in vivo (Fig. 4H) indicating destabilization of the CD147-MCT1 complexes. Additional functions of CD147 include induction of matrix metalloproteinases (MMPs) and vascular endothelial growth factor (VEGF). To investigate whether these functions were impaired, MMP7, VEGFR2 and CD31 stains were performed. The lack of MMP7 expression and the decrease in angiogenesis was evident in the CD147-KD cells when compared to controls, confirmed by reduced CD31 and VEGFR2 histoscores in tumors with CD147-KD (Fig. 4G). There were no differences in proliferation and apoptosis, as shown by MiB1 and caspase 3 activated, respectively (data not shown). These findings further establish CD147 as a functionally relevant protein in ALK+ ALCL cells necessary for survival and tumor growth.

### ALK+ ALCL cells are dependent on CD147-MCT1 transmembrane complexes for glucose metabolism and tumor growth

**Fig. 5:**
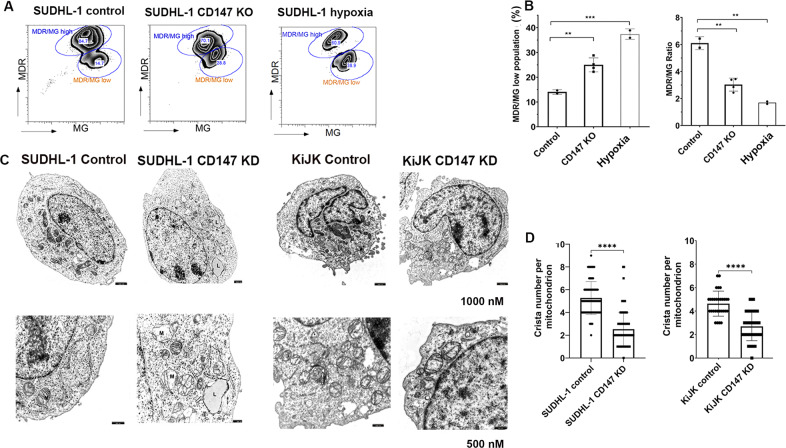
Impairment of CD147-KD and CD147-KO results in mitochondrial damage and accumulation of depolarized mitochondria in ALK+ ALCL cell lines. **A** According to the ratio of MDR to MG staining using FACS analysis, two populations were recognized MDR/MG high and MDR/MG low. Both populations were determined in SUDHL-1 control (CD147 WT *n* = 2), SUDHL-1 cells with CD147-KO (*n* = 4) and in SUDHL-1 control with hypoxia. **B** Bar blots showing the percentage of cells with MDR/MG low staining (left) and the MDR/MG ratio (right) displayed among the groups. A high percentage of low staining cells and reduced MDR/MG ratio, correlates to low mitochondrial activity and membrane potential. Each point represents the average of three biological replicate measurements. Unpaired *t*-test, **p < 0.01, ***p < 0.001. **C** Electron microscopy demonstrates reduced number of mitochondria (M), many swollen with reduction of the number of cristae and cristae-length in ALCL cell lines (SUDHL-1 and KiJK) CD147 KD cells when compared to intact mitochondrial morphology of control cells. Additionally, note the accumulation of lipid vesicles (L) in the cytoplasm of ALK+ ALCL CD147 KD cells. **D** Bar plot displaying the quantification of Crista number per mitochondrion in CD147 WT and CD147KD cells. All mitochondria per cell were counted, (WT, *n* = 10 and KD *n* = 10 per every cell line). Unpaired *t*-test, **p < 0.01, ***p < 0.001.

CD147-MCT1 transmembrane complex has been shown to have a central role in cellular metabolism – particularly glycolysis – a major source of energy production in cancer cells [34]. Accordingly, in vivo PET analysis revealed a remarkably reduced uptake of the glucose analog [^18^F]FDG in CD147-KD KiJK or SUDHL-1 tumors when compared to untreated tumor (Fig. 4A, E). These results indicate that ALK+ ALCL have high-energy consumption and therefore addiction to lactate transport through the CD147-MCT1 complex and point towards increased glycolysis of neoplastic ALK+ ALCL, a phenomenon described as the Warburg effect. To investigate further the impact of CD147 blockade in relation to tumor energy metabolism, the mitochondrial fitness of tumor cells was investigated. Measurement of ΔΨm revealed a loss of mitochondrial depolarization in SUDHL-1 cells with CD147-KO compared to control (SUDHL-1 CD147 WT cells). CD147-KO cells show decreased mitochondrial activity per mitochondrial mass demonstrated by higher percentage of cells with low staining for MDR compared to MG, referred as MDR/MG low population. Conversely, the control cells show higher MDR staining compared to MG referred as MDR/MG ratio (Fig. 5A, B). A similar scenario occurs in SUDHL-1 under hypoxic conditions (Fig. 5A, B). The TEM assay performed in SUDHL-1 and KiJK supported these findings, revealing swollen mitochondria with disrupted cristae structure and reduced mitochondrial cristae number and length (Fig. 5C, D).

**Fig. 6:**
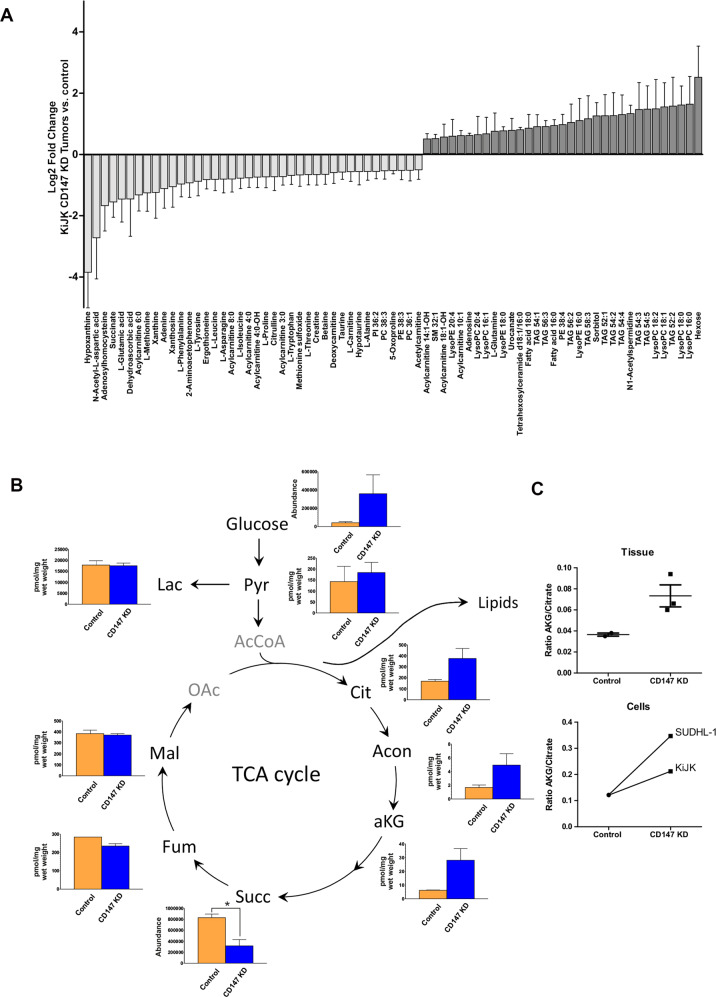
Investigation of metabolic alterations upon CD147-KD in vivo. **A** Non-targeted profiling of metabolites in a mouse xenotransplant model that differ between CD147-KiJK-1 tumors and controls with an absolute log_2_ fold change >0.5 (mean ± SEM, *n* = 2 for controls, *n* = 3 for CD147-KD). **B** Concentrations of glycolytic metabolites and TCA cycle intermediates in CD147-KiJK tumors and controls (mean ± SEM, *n* = 2 for controls, *n* = 3 for CD147-KD). **C** Changed metabolite ratio α-ketoglutarate (AKG) to citrate in CD147- KiJK tumors and controls (mean ± SEM, *n* = 2 for controls, *n* = 3 for CD147-KD) and in SUDHL-1 and KiJK CD147-KD cells compared to controls (*n* = 1 biological replicate per cell line) indicates shift of metabolism to reductive glutaminolysis due to compromised mitochondrial quality. Unpaired *t*-test, *p < 0.05.

For a more detailed investigation, metabolomic analyses both in vivo and in vitro were performed. Initially, exploratory analysis was carried out in vivo by liquid-chromatography mass spectrometry-based non-targeted metabolomics (i.e., metabolomic profiling), a hypothesis-free approach that aims to capture as many metabolites as possible in a single analysis. Metabolomic profiling of SUDHL-1 tumors of mice showed changes of metabolites involved in amino acid metabolism, nucleotide biosynthesis and lipid metabolism (Fig. 6A). Despite reduced glucose uptake, an increased intra-tumoral level of hexose was observed in KiJK + CD147-KD-tumors compared to controls. Higher levels of TAG species are in line with the accumulation of lipid vesicles in vitro observed in TEM (Fig. 5C). Further changes related to lipid metabolism indicated an increase of lyso-phospholipids (e.g., Lyso PC 16:0) and free fatty acids in KiJK + CD147-KD-tumors (Fig. 6A). To assess a potential increased entry of glucose in the TCA cycle, TCA intermediates were quantified with a targeted approach by mass spectrometric methods. These analyses are complementary to the profiling experiment as they capture pre-defined analytes with high accuracy and precision. As a result, concentrations of citrate, aconitate and α-ketoglutarate (AKG) were increased, while TCA intermediates downstream of AKG, such as succinate and fumarate, were found reduced in tumors depleted of CD147 (Fig. 6B). This metabolite pattern hints to an impaired electron transport chain (ETC) and maintenance of TCA cycle by increased entry of glutamine into the TCA cycle with subsequent reductive carboxylation of AKG to citrate. Indeed, the metabolic ratio AKG/citrate as an indicator of reductive glutamine carboxylation [35] increased considerably upon CD147-KD (Fig. 6C). Cellular experiments confirmed these changes albeit to a smaller extent. CD147-KD cells showed higher intracellular concentrations of lactate, pyruvate and TCA cycle intermediates (Supplementary Fig. 8A) and an increase of the AKG to citrate ratio (Fig. 6C). Next, in order to assess a more specific contribution of CD147 to mitochondrial function, we analyzed the OCR as an indicator of mitochondrial respiration by real time metabolic flux measurements in cell culture. Analyses of the OCR in SUDHL-1 and KiJK cells; however, indicated an increased basal and maximal respiration of CD147-KD cells compared to control cells (Supplementary Fig. 8B).

## Discussion

In this study, we aimed to identify the downstream targets of miR-146a, a tumor suppressor miRNA in ALK+ ALCL. We focused on CD147, because we recently reported that this protein is induced by C/EBPβ and it is differentially expressed in ALK+ versus ALK− ALCL cases, indicating a specific role of CD147 in ALK+ ALCL tumor development [33]. This finding was corroborated in a large cohort of ALK+ and ALK− ALCL primary cases (*p* = 0.0001).

**Fig. 7:**
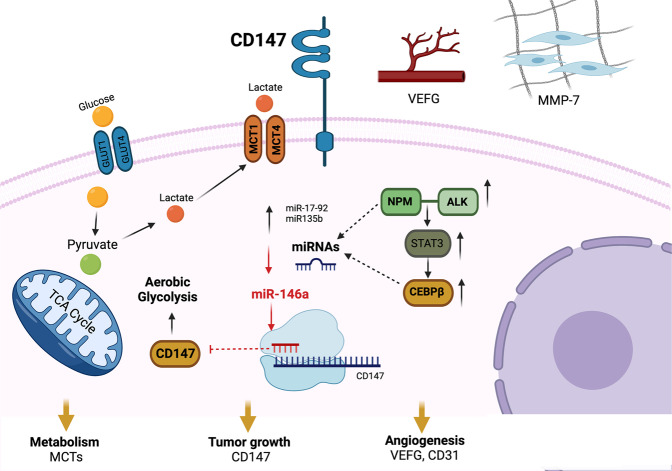
MiR-146a targets CD147 in ALK+ ALCL promoting increased energy metabolism. NPM-ALK promotes CD147 expression by downregulation of miR-146a and overexpression of C/EBPβ through the STAT3 signaling pathway [33]. CD147 promotes tumor growth and invasion by enhancing aerobic glycolysis in ALK+ ALCL.

We now confirmed CD147 as miR-146a direct target gene and demonstrate that functional CD147-MCT1 transmembrane complexes are necessary for cellular metabolism supporting tumor growth, angiogenesis, and invasion in vitro and in vivo in ALK+ ALCL cells (Fig. 7) [36]. Accordingly, CD147 as a direct target of miR-146a has been demonstrated in solid cancers such as NSCLC, HCC and renal cancer [37, 38].

CD147 or extracellular matrix metalloproteinase inducer (EMMPRIN) is usually upregulated in T cells upon activation; however, its expression both at protein and mRNA levels is rather strong in ALK+ ALCL cases when compared with ALK− ALCL cases or normal reactive T cells [33]. CD147 is a transmembrane glycoprotein of the immunoglobulin superfamily expressed in a wide variety of cell types and tumors with pleiotropic functions including development, activation, proliferation, migration, adhesion and invasion, which are important for the pathogenesis of various diseases [33, 39, 40]. Induction of matrix metalloproteinases (MMP) is considered an important oncogenic aspect of CD147 function [40, 41]. Accordingly, the downregulation of CD147 resulted in lack of expression of its downstream target MMP7 and correlated with lack of engraftment of ALK+ ALCL cells or striking tumor growth retardation. These results show that CD147 expression is needed for engraftment and growth of ALK+ ALCL.

A widespread hallmark of cancer cells is an altered energy metabolism, where the cells activate glycolysis to meet their energy demand for rapid proliferation, known as “Warburg effect” [39]. CD147 is an important modulator of the “Warburg effect” by sustaining glycolysis and inhibiting mitochondrial biogenesis and oxidative phosphorylation in tumor cells [42, 43]. A side effect of glycolysis is the accumulation of lactic acid by products. As CD147 also acts as a chaperone for MCT1, it seems to regulate the altered energy metabolism in cells by shuttling MCT1 to the plasma membrane, thereby mitigating the toxic buildup of lactic acid [44, 45]. We now demonstrate that CD147 is essential for ALK+ ALCL tumor viability. Accordingly, PET analysis showed a reduced [^18^F]FDG-uptake after CD147 downregulation, clearly indicating reduced glucose consumption and TAG species accumulation in ALK+ ALCL cells. This is supported by higher intra-tumoral levels of hexose hence pointing to reduced aerobic glycolysis in the absence of CD147. Furthermore, the higher basal respiration rate of CD147-KD cells compared to controls demonstrates a redirection of the metabolism towards mitochondrial respiration, which is in accordance with previous findings in colon adenocarcinoma and glioblastoma [46] as well as in lung cancer cell lines [47].

Non-functional MCT1 might be the cause for the observed mitochondrial morphological changes in the absence of CD147, as mild acidosis has been demonstrated to induce reprogramming of mitochondrial respiratory efficiency including restructuring of the mitochondrial network [48]. The increase in intracellular lactate observed in CD147-KO cells may reflect a condition of low pH with similar consequences on mitochondrial respiration. Elongation or fusion of mitochondria generally occurs in conditions with increased ATP production and represents an adaptive pro-survival response against stress like starvation and has also been associated with cellular senescence [48, 49]. The phenotype described for senescent cells is similar to the changes upon CD147-KD in ALK+ ALCL. Senescent cells show a subpopulation of mitochondria with a lower membrane potential and partial uncoupling of oxidative phosphorylation, which is compensated by an increase of basal respiration [50].

Alterations in TCA cycle metabolites were observed in CD147-KD cells indicating increased reductive carboxylation of glutamine thus maintaining lipogenesis even under hypoxic conditions or respiratory impairment [35, 51]. This is in line with the observed accumulation of lipid vacuoles and increased TAG content in the cytoplasm of ALK+ ALCL cells after CD147-KD. A further reason for enhanced lipid accumulation might be related to the loss of squalene monooxygenase (SQLE), which is characteristic for ALK+ ALCLs and leads to cholesterol auxotrophy and accumulation of squalene [52]. Consequently, the cells become dependent on uptake of exogenous cholesterol by low-density lipoprotein receptor (LDLR) resulting in alterations of the cellular lipid profile. Furthermore, squalene is stored in lipid particles/droplets [53] and is known to affect lipid clustering [54]. Taken together, this might in turn be responsible for an increased lipid uptake and lipogenesis independent of CD147 expression.

CD147 is also known to stimulate the expression of VEGF and MMP in tumor and stromal compartment leading to angiogenesis [40, 55, 56]. The absence of CD147 resulted in reduced expression of VEGFR2 and CD31 in the engrafted tumors, which, in addition to the metabolic effects, likely enhanced growth retardation. This reinforces the role of CD147 in angiogenesis and further supports the hypothesis that metabolic reprogramming and angiogenesis are tightly associated.

Our metabolic analyses suggest that suppression of CD147 might target aberrant glycolysis, thus impairing the major energy source of tumor cells with consequent strong tumor growth restriction. Accordingly, repressing CD147 has been proposed as a novel therapeutic strategy for HCC [57, 58] and malignant melanoma [59]. Interestingly, a recent study showed that immunomodulatory drugs such as thalidomide and its derivatives lenalidomide and pomalidomide act by disrupting the cereblon-CD147-MCT1 axis to exert their antitumor activity, highlighting the importance of the CD147-MCT1 complexes for survival of tumor cells [60].

In conclusion, our data show that low miR-146a expression in ALK+ ALCL results in high CD147 expression. Due to CD147 involvement in multiple tumor-promoting mechanisms, CD147 has the potential to serve as a novel therapeutic target in ALK+ ALCL and warrants further investigation.

## Supplementary information

Supplemental Material
