# Supplementary material for: CD147 a direct target of miR-146a supports energy metabolism and promotes tumor growth in ALK+ ALCL
Source: Leukemia. 2022 Jun 8;36(8):2050–63. doi: 10.1038/s41375-022-01617-x (PMC9343252; doi:10.1038/s41375-022-01617-x)
Supplement: Supplementary file 1 — Supplemental Material [file 41375_2022_1617_MOESM1_ESM.docx]

**Supplemental material**

***Montes-Mojarro and Steinhilber et al***

Supplemental Methods........................................................Page 2

Supplemental Table….........................................................Page 10

Supplemental Figures..........................................................Page 13

**Supplemental Methods**

**Cell culture**

SUDHL-1, KiJK, Karpas 299, SUP-M2, SR-786 were purchased either from the American Type Culture Collection (ATCC) or from Deutsche Sammlung von Mikroorganismen und Zellkulturen (DSMZ). All five ALK+ALCL cell lines have been authenticated and are suitable for in vitro model system for ALCL.[1] The ALK-ALCL cell line Mac-1 was provided by Eva Geißinger (University of Würzburg, Germany) and the ALK-ALCL cell lines Mac2a and FE-PD were obtained from Olaf Merkel (Medical University Vienna, Wien, Austria). The mantle cell lymphoma cell lines (Jeko-1 and Rec-1) were donated by Dr. Dolors Colomer (University of Barcelona, Spain). The carcinoma cell lines (HeLa, HEK293-T), the multiple myeloma cell line (KMS-12), the diffuse large B-cell lymphoma cell line (SUDHL-4), the promyelocytic leukemia cell line (HL-60) and the T-cell acute lymphoblastic lymphoma (Jurkat) were also acquired from ATCC or from DSMZ. All cell lines were regularly tested for mycoplasma contamination.

**T-cells isolation from healthy donors**

CD3+ T cells were isolated from peripheral blood of three healthy individuals using MACS Separation System (Miltenyi Biotec, CA, USA). Pre-enrichment of PBMCs was performed by standard Ficoll density gradient centrifugations. T cells were labeled with CD3 MicroBeads (Miltenyi Biotec, CA, USA) and magnetically separated using VarioMACS.

**Crizotinib treatment**

Crizotinib (PF-02341066) was synthetized and purchased at Med Chem Express (Beutelsbach, Bayern, Germany). Stock solutions were prepared in DMSO solution and diluted in PBS to reach 25, 50 and 100 nM concentrations. ALK+ALCL cell lines (Karpas 299 and SUDHL-1) were incubated in T25 flask at 37°C in the presence of the increasing concentrations of Crizotinib by triplicates. Medium was replenished every 24 hours and fresh medium with antibiotic and corresponding Crizotinib concentration was given. After 72 hours of treatment, cell pellets were retrieved, protein and total miRNA were isolated. ALK inhibition was confirmed and the expression levels of CD147 and miR-146a were evaluated.

**Experimental mice**

Subcutaneous inoculation of 1.5x10^6^ human T-cell lymphoma cells (KiJK or SUDHL-1 or respective cells with CD147-KD) was performed in female 6-8 weeks old NOD scid gamma immune deficient mice (NSG; Charles River Laboratories, Sulzfeld, Germany) under anesthesia with 1.5% isoflurane (Abbott, Wiesbaden, Germany) and 98.5% O_2_. Sample size was determined with the Institute for Clinical Epidemiology and Applied Biometry, University Hospital Tübingen for exploratory study criteria and approved by the Regierungspräsidium Tübingen. No randomization was used. Animals were excluded of the study in case of suffering (e.g., feeding instability, cachexia, weight loss), signs of toxicity or intolerance of the treatments, 20% body weight loss or ulceration of the skin. Investigators were blinded to the group for all further assessments including: tumor engraftment, tumor growth and tumor immunohistochemistry evaluation.

**RNA isolation**

Total RNA was extracted from formalin- fixed paraffin-embedded tissues in primary cases using phenol/chloroform extraction followed by DNase treatment (DNA-free™ Kit, Applied Biosystems, Ambion, Carlsbad, CA, USA), as described elsewhere [2].

**Overexpression of miR-146a**

SUDHL-1 or Karpas 299 cells were seeded at a concentration of 1.8x10^5^ onto 24-well plates and were transfected with miRNA Mimics (400 nM end concentration) using 3 µl HiPerFect Transfection Reagent (Qiagen) in a total of 180 µl RPMI according to the manufacturer’s protocol. For miRNA or RNA analysis the cells were harvested 32 h after transfection. For protein analysis the cells were transfected a second time 72 h after first transfection and cells were harvested 24 h after second transfection. The miRIDIAN microRNA Mimic Negative Control #1 was used to exclude undesirable side effects through the transfection procedure. The Mimic Housekeeping Positive Control #2 (GAPD) was overexpressed to confirm the GAPDH downregulation on mRNA level.

**Mature miRNA quantification**

Mature miRNA quantification, cDNA synthesis and RT-qPCR analysis were performed as previously described using miScript Reverse Transcription Kit, miScript Primer assays Hs_miR-146a_1 and miScript SYBR Green PCR Kit (Qiagen) [3].

MiRNA expression was normalized to miR-106b, a miRNA homogenously expressed in ALCLs, using miScript Primer assay Hs_miR-106b_1 (Qiagen). The data were analyzed as previously described [4].

**Transcriptome analysis**

Transcriptome analysis using NGS was performed by CeGaT (Tübingen, Germany). RNA quality was checked with a Bioanalyzer (Agilent Technologies, Santa Clara, CA, USA) using the Agilent RNA 6000 Nano Kit according to the manufacturer’s instructions. Using the RNase-Free DNase Set (Qiagen), 1 µg RNA was digested. RNA was purified with the RNeasy MinElute Cleanup Kit (Qiagen), before RNA quality was checked again. Ten ng RNA were used for cDNA synthesis with the Ovation RNA-Seq System V2 (NuGEN, San Carlos, CA, USA), before quality was tested with the RNeasy MinElute Cleanup Kit (Qiagen). The libraries were amplified with Herculase II Fusion DNA Polymerase (Agilent Technologies) and Sure Select Primers with four PCR cycles. Indexes were added with Herculase II Fusion DNA Polymerase (Agilent Technologies) and SureSelect Indexing Primers with 8 cycles. Both amplifications were conducted with SureSelect Library Prep Kit and SureSelect Target Enrichment Kit. Final quality control was performed with the Agilent DNA 1000 Kit and a Bioanalyzer. For paired-end transcriptome sequencing the 2500 sequencing system (Illumina, San Diego, CA, USA) was applied. For data preprocessing first raw sequence files were processed and de-multiplexed with CASAVA1.8.2 (Illumina). Then adapter sequences were removed with cutadapt 1.2.1. Reads were aligned to human transcriptome (hg19) with TopHat (v.2.0.8b). Normalized gene expression (FPKM) in samples was calculated as well as the logarithmic fold change (cutdiff 2.1.1) between samples x and y. Value >0 means, that sample x has a higher expression level and value <0 means, that sample y has a higher expression level. Enrichment of manually ranking list was calculated using Gene Set Enrichment Analysis (GSEA, V4.1.0) by the preranked analysis option. DESeq2 log_2_ fold change values between untransfected and cells with mi-R146a overexpression were used to rank the genes.

**Real-time quantitative RT-PCR**

cDNA synthesis from RNA and real-time quantitative RT-PCR analysis (RT-qPCR) to quantify the mRNA level of CD147 was performed using Universal Probe Library (UPL) assays (Roche Applied Science, Penzberg, Germany) (Forward-Primer: GGGAGAGTACTCCTGCGTCTT, Reverse-Primer: ACTTCACAGCCTTCACTCTGG, Probe 42) and *ACTB* as housekeeping gene, the LightCycler 480 Probes Master and the LightCycler 480 System for detection (Roche). Mature miRNA quantification, cDNA synthesis and RT-qPCR analysis were performed as previously described [3].

**Western Blot analysis**

For immunoblotting we used the CD147 (BSG) antibody (HIM6, 555961) (BD Biosciences, San Diego, CA, USA), MCT1 (H1, SC365501) (Santa Cruz Biotechnology, Heidelberg, Germany) Phospho-Stat3 (Tyr705) (3E2, 9138), Phospho STAT1 (Tyr701, 9167S), Phospho-Stat3 (Tyr705,) both from Cell Signaling (Beverly, MA, USA); ALK (45CB8,354300) (Thermo Fisher Scientific (Waltham, MA, USA), CD30 (M0751/BerH2; Dako/Agilent, Santa Clara, CA, USA) and α-Tubulin (T5168) (Sigma-Aldrich, Steinheim, Germany) as loading control.

**Cloning of CD147shRNA sequences**

Five different oligonucleotides containing the CD147-shRNA following sequences were cloned into pFUGW as previously described [4]: CD147-shRNA A (5′ AGCTTTTCCAAAAAGT CGTCAGAACACATCAACTCTCTTGAAGTTGATGTGTTCTGACGACGGG 3′), CD147-shRNA B (5′ TGACAAAGGCAAGAACGTCTCTCTTGAAGACGTTCTTGCCTTTGTCA 3′), CD147-shRNA C (5′AGCTTTTCCAAAAAGGTTCTTCGTGAGTTCCTCTCTCTTGAAGAGGA ACTCACGAAGAACCGGG 3′), CD147-shRNA D (5′ AGCTTTTCCAAAAAGCTACACATT GAGAACCTGAATCTCTTGAATTCAGGTTCTCAATGTGTAGCGGG 3′), CD147-shRNA E (5′ AGCTTTTCCAAAAAGGCTGTGAAGTCGTCAGAACATCTCTTGAATGTTCTGACGACTTCACAGCCGGG 3′). To achieve efficient knockdown (KD) of *CD147*, infection was repeated after 24 h (MOI of 90).

**CD147 shRNA knockdown**

Five different shRNAs were tested for most efficient knockdown of CD147. Specific shRNA sequences had been published [5, 6] or were designed using the BLOCK-iT™ RNAi Designer (ThermoFisher Scientific) online tool. Oligonucleotides were cloned as previously described [7]. To test for shRNA efficiency pSuper vector shRNA constructs were transfected into HeLa cells. Briefly 1x10^6^ cells were seeded in 5 ml Dulbecco’s modified eagles medium with 10% FCS, 100 µg/ml penicillin, and 100 µg/ml streptomycin (ThermoFisher Scientific) in 6 cm plates. After 24 hours shRNA constructs were transfected using Opti-MEM, 4 µg plasmid and 10 µl Lipofectamine (ThermoFisher Scientific) following the manufacturer’s instructions. After 72 hours cells were harvested and mRNA was isolated using the RNeasy mini kit according to the manufacturer’s protocol (Qiagen, Hilden, Germany). RT-qPCR was performed as stated in the materials und methods section of the manuscript.

**CD147 Knockout using CRISP/Cas-9 and validation by targeted NGS.**

The sgRNA targeting CD147 gene (CAGGCCCAGTCAGTGACAGG) was designed using online platform (http://guides.sanjanalab.org) [8]. In order to produce the lentivirus, plasmid was co-transfected with the packaging plasmids pVSVg (AddGene 8454) and psPAX2 (AddGene 12260) into HEK293 T cells. Infection was performed in ALK+ALCL cell line (SUDHL-1), and Knockout (KO) was confirmed by WB. Pathogenic mutations leading to CD147 protein damage were investigated with targeted NGS as previously reported. In order to confirm the CD147 knockout, pathogenic mutations leading to CD147 protein inactivation were investigated using targeted NGS analysis. CD147 knockout cell clones were sequenced on the Ion Torrent Personal Genome Machine (PGM) (Thermo Fisher Scientific, South San Francisco, CA, USA). Mutations were studied using the fusion method (Life Technologies, Thermo Fisher Scientific). Design of the primers was carried out with the freely available online program Primer3web (version 4.0.0). The target regions were amplified using the primers forward (5’ GGTTCCAGGCTCCTCTCTC 3’) and reverse (3’ CTCCTTGGCTTCTCACCTTG 5’) were used in order to amplify the CD147 region of interest in a standard PCR. The reads were analysed in the freely available program Integrative Genomics Viewer (IGV, Broad Institute) [9].

**Luciferase reporter assay**

For amplification the primers CD147_F: (AATGGAGCTCAGGTGGCCCGAGGA) and CD147_R (TCTACCTGCAGGGAGTCGAACACAGACCCGTGG) and SRPRB_F: (TTAGTTGTTTAAACGAGGAAGGGGTACAAGATGT) and SRPRB_R: (GACTCGAGGCTAGCGAGACAGGCTGTTTTCTTTAC) were used. The PCR products were inserted into the pmirGLO Dual-Luciferase miRNA target expression vector (Promega, Madison, WI, USA) by using the Rapid DNA Dephos & Ligation Kit (Merck KGaA, Darmstadt, Germany) and the In-FusionTM Advantage PCR Cloning Kit (Clontech Laboratories, Mountain View, CA, USA). Efficient insertion was confirmed by sequencing with primers pmirGlo_F (TGACCGGCAAGTTGGACGCC) and pmirGlo_R (GGCCGCCCCAAGGGGTTATG). For luciferase reporter assays, HEK293T and HeLa cells were cultured in 12-well plates and each well was transfected with 2 µg pmirGLO Dual-Luciferase miRNA target expression vector and 16 µl miRIDIAN microRNA mimic 146a using 6 µl Lipofectamine 2000 (Invitrogen). Cells were assayed in triplicates and in two independent experiments using Dual-Luciferase® Reporter Assay System (Promega) 40 h after transfection.

**Sequential PET/MRI**

Approximately 13±2 MBq of [^18^F]FDG was injected intravenously in a volume between 50-100 µl 0.9% NaCl. After an uptake time of 60 min under anesthesia, mice were scanned on an Inveon Small Animal PET-Scanner (Siemens Preclinical Solutions, Knoxville, Tennessee, USA) for 10 min. Subsequently, the mice were transferred to a 7T BioSpec MRI scanner (Bruker BioSpin MRI GmbH, Ettlingen, Germany). The MR-scan was performed using a rat whole body coil and a T2-weighted 3D TurboRARE sequence (repetition time = 1800 ms; echo time = 66.7 ms; 0.3 mm resolution; averages: 2).

For PET-image reconstruction a 2D ordered-subsets expectation maximation (OSEM) algorithm with four iterations was applied using Inveon Acquisition Workplace (Siemens Preclinical Solutions) which results in a pixel size of 128x128 and a matrix size of 0.79x0.79 mm^2^. The PET/MRI scans were fused and analyzed in Inveon Research Workplace (Siemens Preclinical Solutions). Volumes of interest (VOI) were drawn on the tumors and the % injected dose per cm^3^ (%ID/cm^3^) to quantify [^18^F]FDG-uptake was calculated.

**Immunohistochemistry:**

Xenograft tumors were fixed in 4% formalin and paraffin embedded. For histology 3-5 µm-thick sections were cut and stained with haematoxylin and eosin (H&E). The slides were stained with ALK1, CD30, CD31 (all from DakoCytomation, Glostrup, Denmark), phospho-Stat3 (Tyr705/3E2) and VEGFR2 (55B11) (both Cell Signaling Technology, Frankfurt, Germany), MMP7, CD147 (78106) (both from Abcam, Cambridge, UK) and MCT1 (H1) (Santa Cruz Biotechnology, Heidelberg, Germany). Appropriate positive and negative controls were used to confirm the adequacy of the staining. Primary ALCL cases were completely immunophenotyped, as part of the diagnostic work-up and classified according to the World Health Organization classification in ALK+ and ALK- ALCL. CD147 Immunohistochemistry was performed and quantified using the histoscore as described below. The staining was scored according to both intensity and the cell percentage with membranous staining. Intensity was assessed as 0 negative, 1+ weak 2+ moderate, and 3+ strong. Histoscore was then calculated by multiplying the intensity of the stains by the percentage of positively stained cells. CD31 and VEFGR2 histoscore was assessed by considering the grading scale described in Supplemental Figure 7. Histoscore was in this case calculated by multiplying the vessels scale (low=1, moderate=2 and high=3) by the percentage of tumor area presenting the scale grade, rendering to a histoscore scale from 0 to 300.

**Transmission Electron Microscopy (TEM):**

Paraffin embedded cell blocks of SUDHL-1 and KiJK with CD147-KD and their respective controls were deparaffinated and fixed using 2.5% Glutaraldehyde (Electron Microscopy Science, Hatfield, PA, USA) in sodium cacodylate buffer (Merck, Darmstadt, Germany) as previously described in detail [10] Embedding in Araldite (Serva, Heidelberg, Germany) was performed by the embedding machine, Leica EM TP (Leica, Wetzlar, Germany). Finally, the samples underwent polymerisation at 60°C.Ultrathin sections (50-70nm) were cut by ultramicrotome (Leica, Wetzlar, Germany) with a diamond knife. Lastly, ultrathin sections were mounted on copper grids. TEM images were acquired with an EM10 electron microscope (Carl Zeiss, Oberkochen, Germany) and a digital camera (Tröndle, Germany).

**Non-Targeted metabolomic profiling:**

In brief, frozen xenograft tumors were subjected to homogenization in methanol/water (1:1, v/v) followed by lipid extraction with methyl *tert*-butyl ether (MTBE)/methanol (3:1, v/v). Dried aqueous and dried lipid extracts were reconstituted at a tissue/solvent ratio of 0.03 mg/µl in acetonitrile:water (95:5, v/v) and isopropanol:methanol (3:1, v/v), respectively. Aqueous extracts were analyzed after HILIC separation and lipid extracts were analyzed after RPLC separation, respectively.

**Targeted Metabolomics**

Aliquots of the tumor tissue homogenates in methanol/water corresponding to about 1 mg of tissue were used for analysis. Tissue measurements were performed in duplicate. Cell pellets (1 mio cells) were extracted with 200 µL of methanol:acetonitrile:water (2:2:1, v/v), precooled to -20 °C, as described, and 10 µl or 20 µl aliquots were used for quantification of lactate, or TCA cycle intermediates, respectively.

**XF Cell Mito Stress Test using Seahorse XFe96 Analyzer**

Briefly, each well of a XF 96-well cell culture plate was coated with Cell-Tak, following the manufacturer’s basic absorption coating protocol. Cells were settled down for Seahorse XFe96 analysis using centrifugation following the protocol for immobilization of non-adherent cells for assay on the Agilent Seahorse XFe96 Cells were seeded at a density of 7.5 x 10^4^ cells in each well of a XF 96-well cell culture plate, in 50 µl pre-warmed assay medium (Seahorse XF Base Medium supplemented with 10 mM glucose (Sigma Aldrich Chemie), 2 mM glutamine (Biozym Scientific GmbH) and 1 mM sodium pyruvate (Fisher Scientific) pH 7.4). OCR rates and the relative levels of basal as well as maximal respiration were calculated using Seahorse Wave software (Agilent).

**Measurement of mitochondrial membrane potential (ΔΨm) using FACS analysis**

SUDHL-1 cells untreated (CD147 WT), SUDHL-1 cells with CD147-KO and SUDHL-1 cells under stress conditions (6 days starvation) were treated with or without 10 µM Oligomycin A (Sigma Aldrich) for 2.5 hours. Cells in culture were split every two days using RPMI 1640 GlutaMAX I (supplemented with 10% fetal calf serum, 2 mM glutamine, 100 μg/mL penicillin and 100 μg/mL streptomycin (Thermofisher Scientific). For the cells under starvation condition a fasting mimic medium comprised of glucose-free RPMI (Gibco, USA) was used. After incubation, the cells were stained with 100 nM Mito Tracker Green (MG) and 10 nM Mito Tracker Deep Red (MDR) (ThermoFisher Scientific) during 15 minutes in a humified incubator chamber at 37°C with 5% CO_2_ [11]. FACS analysis were performed by FACS Calibur flow cytometer (Becton Dickinson) and data were analyzed using FlowJo V9.9.6.

**Statistical Analysis:**

Descriptive statistics were used to describe the data. Continuous variables were expressed as mean and standard deviation (SD) or median and interquartile range according to their distribution. The distribution was evaluated using Shapiro-Wilk test and by examining kurtosis, skewness, and Q-Q plots and histograms. Furthermore, the data are presented as bar plots and boxplots and/or as mean ± standard error of the mean (SEM) of a representative experiment in triplicate using Prism Graph-Pad8. Bivariate analysis was performed, to compare two groups Student's t-test was used for continuous normally distributed variables, while the Wilcoxon rank sum test was used to evaluate skewed variables. One-way analysis of variance (ANOVA) was used to compare continuous variables in more than two groups. Before running the model, several assumptions were tested (Normal distribution of residuals and homoscedasticity by Levene's test). Pairwise comparison was done using Bonferroni correction. All reported p values were two-sided and the significance level was set at ≤0.05. All the analyses were carried out using the statistical program for social sciences IBM SPSS software version 27.0 (IBM, New York, NY, USA) and Prism Graph-Pad8.

**Supplemental Tables**

**Supplemental Table 1.Genes differentially downregulated by miR146a**

| **GENE** | **LOCUS** | **Log^2^ Fold Change** | **p-Value** | **FDR-q** |
| --- | --- | --- | --- | --- |
| ***PSENEN*** | **chr19:36236493-36237903** | **3,94355** | **5,00E-05** | **0,01025** |
| ***ZNF275*** | **chrX:152599612-152618384** | **1,25774** | **5,00E-05** | **0,01025** |
| ***SRPRB*** | **chr3:133502876-133540336** | **1,17778** | **5,00E-05** | **0,01025** |
| ***PNPO*** | **chr17:46018888-46026674** | **1,0203** | **0,0002** | **0,0301732** |
| *BRK1* | chr3:10157332-10168874 | 1,03499 | 0,0005 | 0,0539474 |
| *GINS1* | chr20:25388322-25429191 | 1,0414 | 0,00085 | 0,0748043 |
| *SET* | chr9:131445933-131458675 | 0,804718 | 0,001 | 0,0845874 |
| *IL31RA* | chr5:55147206-55218682 | 1,13278 | 0,00105 | 0,087125 |
| *TRAF6* | chr11:36505316-36531863 | 1,18981 | 0,00125 | 0,0963772 |
| *HIST1H2BM* | chr6:27782821-27783267 | 0,987399 | 0,0014 | 0,1047 |
| ***PIK3AP1*** | **chr10:98353068-98480279** | **0,926083** | **0,0015** | **0,109821** |
| *GZMB* | chr14:25100160-25103432 | 0,88632 | 0,00195 | 0,128952 |
| *HNRNPD* | chr4:83274466-83295149 | 0,771246 | 0,0025 | 0,152583 |
| *SLC38A10* | chr17:79218798-79269096 | 1,25347 | 0,0029 | 0,167882 |
| *TMEM194A* | chr12:57449425-57472574 | 0,902114 | 0,00305 | 0,172273 |
| *GOLPH3L* | chr1:150618700-150669672 | 0,964231 | 0,00315 | 0,17425 |
| *TMX1* | chr14:51706885-51724372 | 0,849133 | 0,00315 | 0,17425 |
| *ZNF691* | chr1:43312243-43318146 | 1,43513 | 0,0036 | 0,190669 |
| *FBXL3* | chr13:77579388-77601331 | 0,98655 | 0,0046 | 0,216051 |
| *MLLT1* | chr19:6210391-6279959 | 0,8022 | 0,0046 | 0,216051 |
| *PHKB* | chr16:47495209-47735434 | 0,902275 | 0,00465 | 0,216938 |
| *DNAJC6* | chr1:65730376-65881552 | 1,2861 | 0,00495 | 0,224912 |
| *SH3BP5* | chr3:15295690-15382901 | 0,796296 | 0,005 | 0,226593 |
| *SLC10A3* | chrX:153715649-153719002 | 1,20137 | 0,00505 | 0,226794 |
| *PTPN14* | chr1:214522038-214725024 | 0,848826 | 0,0052 | 0,229975 |
| *RAB33B* | chr4:140374960-140397069 | 1,20836 | 0,00555 | 0,237322 |
| *MED20* | chr6:41873091-41888877 | 0,983319 | 0,0057 | 0,239909 |
| ***BSG/CD147*** | chr19:571276-583493 | 0,707638 | 0,0058 | 0,242653 |
| *MORN4* | chr10:99374309-99393913 | 1,61653 | 0,0061 | 0,250986 |
| *ABHD2* | chr15:89631380-89745591 | 0,780464 | 0,0063 | 0,255 |
| *TUT1* | chr11:62342516-62359109 | 0,994844 | 0,00635 | 0,255835 |
| ***CASP2*** | chr7:142985307-143004789 | 0,758672 | 0,00695 | 0,268523 |
| *TNFSF14* | chr19:6663147-6670599 | 1,81462 | 0,00715 | 0,273521 |
| *SCP2* | chr1:53392900-53517289 | 0,828911 | 0,00865 | 0,298468 |
| *C3orf17* | chr3:112721291-112738555 | 0,729022 | 0,009 | 0,30481 |
| *NLRP7* | chr19:55434876-55458873 | 0,85868 | 0,0092 | 0,308585 |
| *MRM1* | chr17:34958024-34965407 | 2,4192 | 0,00935 | 0,312114 |
| *FAM114A2* | chr5:153371268-153418497 | 0,999661 | 0,0095 | 0,31501 |
| *ZNF185* | chrX:152082985-152142025 | 2,89498 | 0,01 | 0,323284 |
| *IRF4* | chr6:391738-411443 | 0,640881 | 0,0101 | 0,324111 |
| *AGAP6* | chr10:51748077-51770259 | 0,915632 | 0,0106 | 0,333704 |
| *ZNF138* | chr7:64254765-64314178 | 0,827329 | 0,0106 | 0,333704 |
| ***ADAM17*** | chr2:9629410-9695917 | 0,734827 | 0,0106 | 0,333704 |
| *LMO7* | chr13:76194569-76434006 | 1,16808 | 0,0109 | 0,336461 |
| *CBX5* | chr12:54624730-54673915 | 0,649145 | 0,01095 | 0,336567 |
| *GZMA* | chr5:54398473-54406080 | 0,677049 | 0,01105 | 0,336914 |
| *PTPRCAP* | chr11:67202980-67205153 | 0,901055 | 0,01155 | 0,344032 |
| *CCDC78* | chr16:771141-776473 | 1,88591 | 0,0121 | 0,348787 |
| *LINC00265* | chr7:39773166-39834222 | 0,852222 | 0,01245 | 0,351594 |
| *TLR6* | chr4:38825328-38858438 | 0,852634 | 0,01265 | 0,354383 |
| *LRRC8C* | chr1:90098643-90185094 | 0,791485 | 0,0131 | 0,360328 |
| *UBXN11* | chr1:26608772-26647014 | 1,04585 | 0,0132 | 0,361935 |
| *RPGRIP1L* | chr16:53633817-53737771 | 0,779499 | 0,01325 | 0,362736 |
| *PTPN22* | chr1:114356432-114447741 | 0,929166 | 0,0133 | 0,363248 |
| *LOC100271836* | chr16:21458003-21513602 | 1,20026 | 0,01365 | 0,368762 |
| *CACTIN* | chr19:3607244-3626813 | 0,991146 | 0,01375 | 0,36889 |
| *KCNC3* | chr19:50818764-50832634 | 0,848814 | 0,01395 | 0,370547 |
| *CARD16* | chr11:104912052-104916051 | 1,16099 | 0,01425 | 0,373112 |
| *EFCAB13* | chr17:45401326-45518677 | 0,877313 | 0,0146 | 0,378016 |
| *SLC45A3* | chr1:205626980-205649630 | 0,962116 | 0,0148 | 0,379809 |
| *TMEM185B* | chr2:120975046-120980984 | 0,677109 | 0,0151 | 0,383274 |
| *UBE2D4* | chr7:43966034-44058748 | 1,4355 | 0,01515 | 0,383984 |
| *BGLAP* | chr1:156182778-156217908 | 0,804297 | 0,01545 | 0,388479 |
| *MCCC2* | chr5:70883114-70954530 | 0,650341 | 0,01545 | 0,388479 |
| *ARID3B* | chr15:74833547-74890472 | 0,694635 | 0,0155 | 0,389456 |
| *FHL1* | chrX:135228860-135293518 | 2,98153 | 0,0159 | 0,395514 |
| *DDX58* | chr9:32455299-32526322 | 0,993179 | 0,0161 | 0,397651 |
| *UGDH-AS1* | chr4:39529458-39640481 | 0,654441 | 0,0168 | 0,404896 |
| *DISP1* | chr1:222988430-223179337 | 1,15611 | 0,0173 | 0,412384 |
| *FBXL17* | chr5:107194733-107717799 | 1,1163 | 0,0175 | 0,414881 |
| *VARS2* | chr6:30881981-30894235 | 0,853045 | 0,0177 | 0,416506 |
| *CACNA2D3* | chr3:54156692-55108584 | 0,805801 | 0,01775 | 0,4174 |
| *MCM8* | chr20:5931297-5975831 | 0,643223 | 0,0181 | 0,419257 |
| *FKBP1A* | chr20:1290554-1373816 | 0,580914 | 0,0182 | 0,419257 |
| *SAMSN1* | chr21:15857548-16015428 | 0,898237 | 0,01845 | 0,4208 |
| *HIST1H3F* | chr6:26250369-26250835 | 0,719557 | 0,01885 | 0,423548 |
| *ZFP62* | chr5:180274610-180288286 | 0,798275 | 0,01895 | 0,424973 |
| *GEN1* | chr2:17935176-17966632 | 0,740877 | 0,01905 | 0,425299 |
| *FBXL13* | chr7:102453307-102715288 | 1,01445 | 0,0195 | 0,430111 |
| *DPP3* | chr11:66247483-66277130 | 0,758752 | 0,01955 | 0,430125 |
| *SYNPO* | chr5:149980641-150038792 | 0,657916 | 0,0201 | 0,432933 |
| *MRPS33* | chr7:140705960-140714781 | 0,951888 | 0,0212 | 0,445317 |
| *SGK196* | chr8:42948656-42978323 | 0,86618 | 0,0214 | 0,447116 |
| *ITGA3* | chr17:48133339-48167849 | 0,617392 | 0,02155 | 0,449173 |
| *NHEJ1* | chr2:219940045-220025587 | 0,798002 | 0,0216 | 0,449677 |
| *FLJ14186* | chr4:120326677-120331815 | 0,661037 | 0,02185 | 0,45245 |
| *SHISA9* | chr16:12995476-13334273 | 0,692088 | 0,022 | 0,454745 |
| *DET1* | chr15:89055713-89089912 | 1,20865 | 0,0221 | 0,456001 |
| *NCOA7* | chr6:126102306-126253176 | 0,59909 | 0,02215 | 0,456492 |
| *NFE2* | chr12:54685890-54694821 | 1,27465 | 0,02225 | 0,458011 |
| *RPP25L* | chr9:34610481-34612110 | 0,924259 | 0,02285 | 0,463787 |
| *LY6G5B* | chr6:31638727-31640227 | 0,79686 | 0,02305 | 0,466488 |
| *PPIL2* | chr22:22020272-22090071 | 0,749354 | 0,02345 | 0,469135 |
| *APMAP* | chr20:24943579-24973425 | 0,72169 | 0,0236 | 0,470784 |
| *FZD3* | chr8:28351721-28431785 | 0,702407 | 0,02385 | 0,473333 |
| *SNRNP27* | chr2:70121074-70132368 | 0,770988 | 0,02395 | 0,474688 |
| *PLAC8L1* | chr5:145463875-145483946 | 1,79154 | 0,02425 | 0,476926 |
| *TIGD7* | chr16:3348807-3368576 | 1,58265 | 0,0242 | 0,476926 |
| *GLUD1P3* | chr10:75491298-75495367 | 1,31234 | 0,02485 | 0,481659 |
| *ZNF773* | chr19:58011308-58019510 | 1,4372 | 0,0249 | 0,482092 |
| *LRRC45* | chr17:79981279-79989027 | 1,76534 | 0,02515 | 0,484509 |
| *AEN* | chr15:89164526-89175512 | 0,699203 | 0,0252 | 0,485204 |
| *IRAK1* | chrX:153275956-153285342 | 0,657034 | 0,02545 | 0,485724 |
| *SDHC* | chr1:161284165-161337673 | 0,598911 | 0,02545 | 0,485724 |
| *PRKCQ-AS1* | chr10:6622386-6627323 | 1,16437 | 0,0255 | 0,486191 |
| *SLC2A3* | chr12:8071823-8088892 | 0,568425 | 0,02565 | 0,486345 |
| *PRSS27* | chr16:2762422-2770552 | 2,17467 | 0,0257 | 0,486499 |
| *NCOA5* | chr20:44689625-44718580 | 0,622129 | 0,0257 | 0,486499 |
| *MRPL19* | chr2:75873908-75889334 | 0,608319 | 0,02585 | 0,488278 |
| *DOCK2* | chr5:169064250-169510386 | 0,609534 | 0,0265 | 0,495453 |
| *NBPF1* | chr1:16890411-16939982 | 0,821834 | 0,0267 | 0,497059 |
| *ANKRD18B* | chr9:33524410-33573001 | 0,791147 | 0,0267 | 0,497059 |
| *NPAT* | chr11:108028118-108093365 | 0,638405 | 0,0274 | 0,504166 |
| *Genes in bold were selected for further validation. | | | | |

**Supplemental Figures**


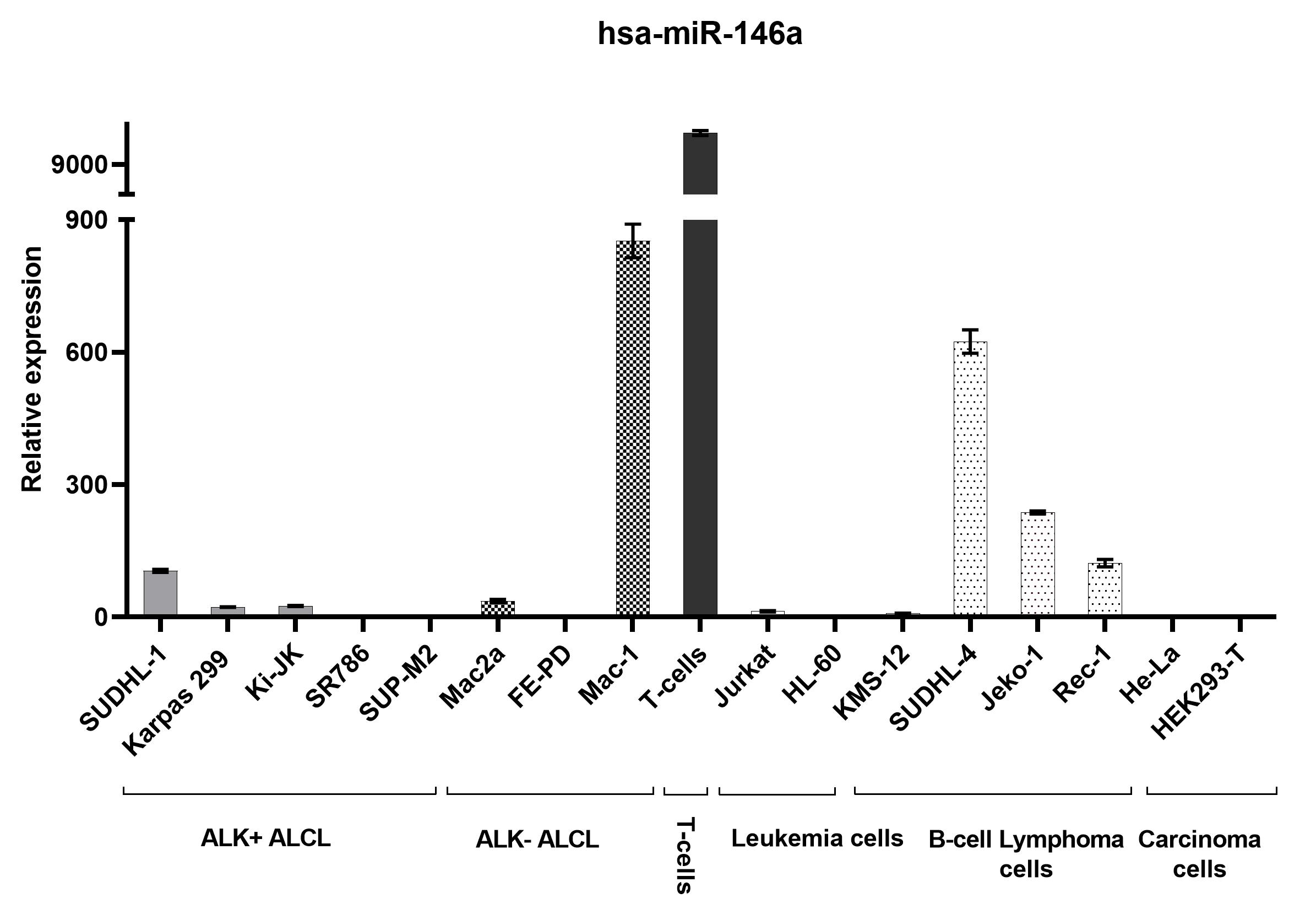


**Supplemental Figure 1.** Comparison of miR146a expression levels in ALK+ALCL cells (SUDHL-1, Karpas 299, KiJK, SR786, SUP-M2), ALK-ALCL cells (FE-PD, Mac-1 and Mac2a), CD3+ T cells from 3 healthy donors, leukemia cells (Jurkat and HL-60), B-cell lymphoma cells (SUDHL-4, jeko-1, Rec-1 and KMS-12) and carcinoma cell lines (HeLa, HEK293-T). Every plot represents the mean ± SD of three independent measurement. For RT qPCR quantifications values were normalized to miR-106b and analyzed using the 2^-ΔΔCp^ method.


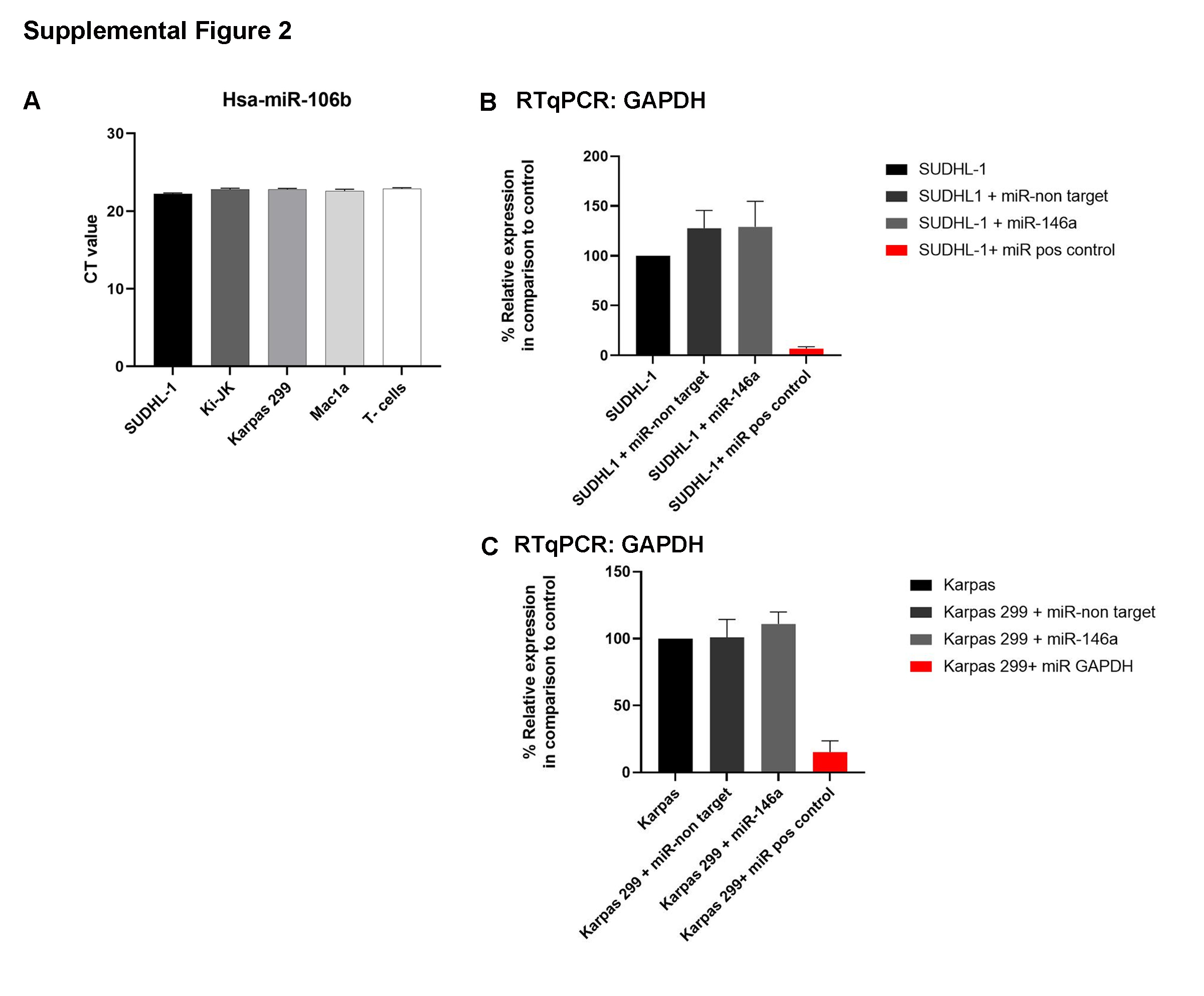


**Supplemental Figure 2A. miRNA expression in ALCL cell lines**. **A**. RTqPCR analysis of miR-106b expression levels (CT values) in different ALCL cell lines. **B and C**. Transfection efficiency of miRNA transient transfection. miR-positive control targets GAPDH. Data represent average from biological triplicates, each bar blot represents the median and standard deviation (SD).**
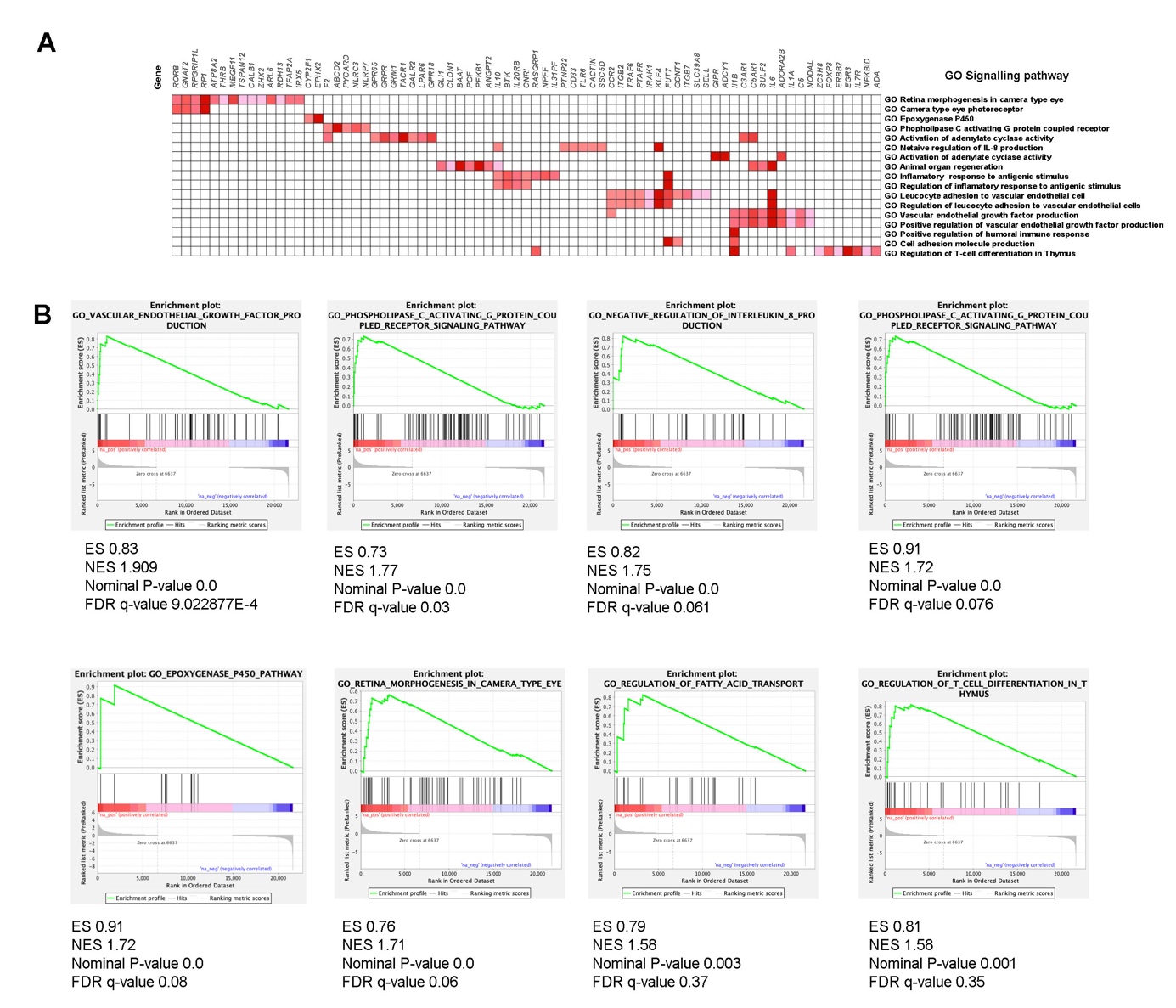
**

**Supplemental Figure 3.** Enrichment of miR-146a differentially expressed genes calculated by GSE. **A.** Hit map of the enriched genes involved in the Gene Ontology (GO) Signaling pathways **B.** Enrichment plots using GSEA of indicated signatures from the ranked list of gene expression profile of ALK+ALCL cells with and without miR-146a overexpression. Enrichment score (ES), Normalized Enriched score (NES).


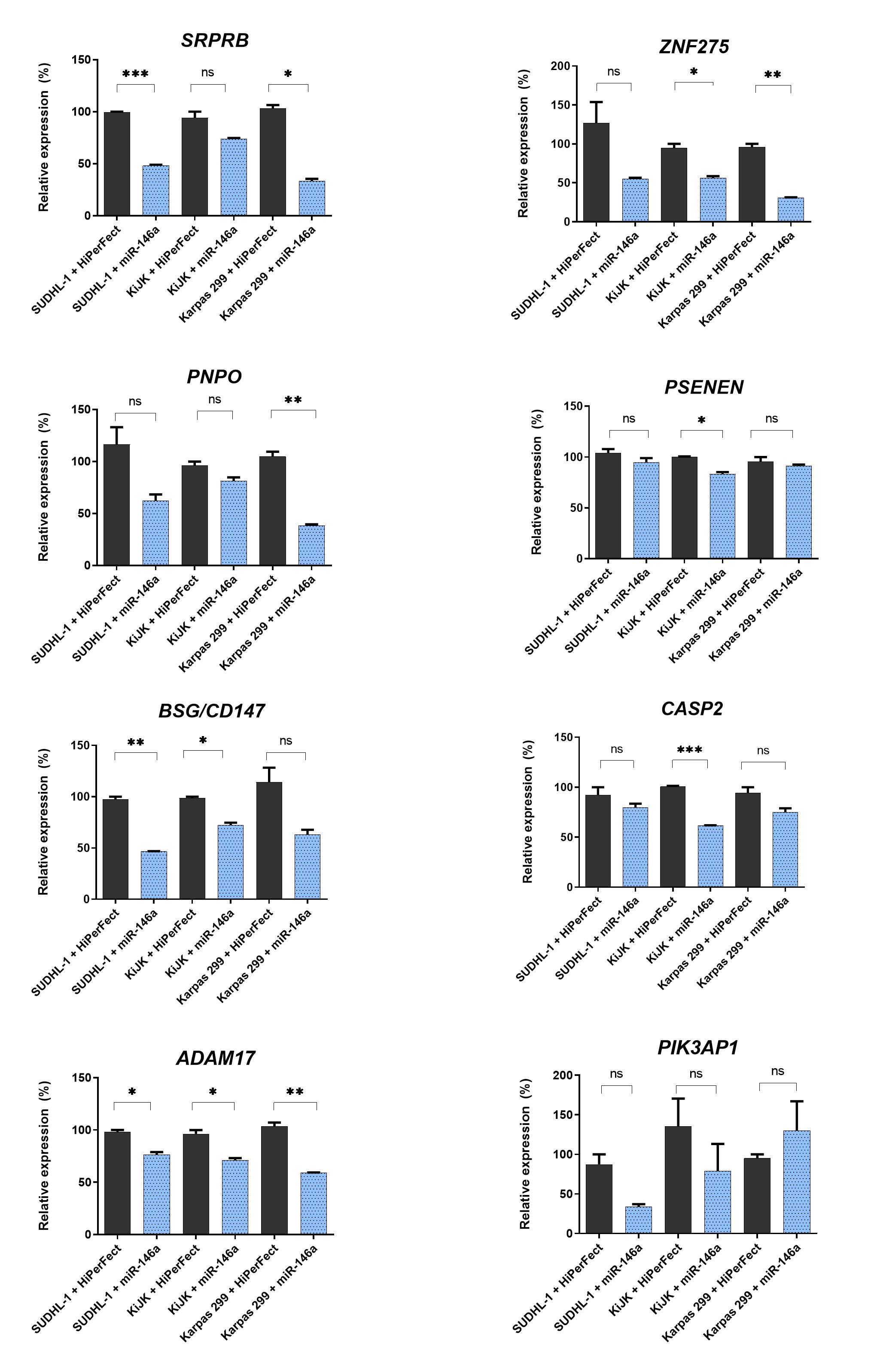


**Supplemental Figure 4.** miR-146a target genes validation. RT-qPCR analysis of miR-146a regulated target genes. Relative expression levels of the eight selected candidate genes are illustrated (*SRPRB*, *ZNF275*, *PNPO*, PSENEN, *CD147, CASP2, ADAM17, PIK3AP1*). Regulation by miR-146a was determined by RT-qPCR analysis in miR-146a transfected or untransfected SUDHL-1, KiJK and Karpas 299 cells. Relative mRNA downregulation of the candidate genes was identified using RT-qPCR quantification, values were normalized to ACTB and GAPD genes, data were analyzed according to the 2^-ΔΔCp^ method. Results are depicted as mRNA levels relative to mean value levels of HiperFect treated cells. Unpaired t-test, *p<0.05, **p<0.01, ***p<0.001.

**
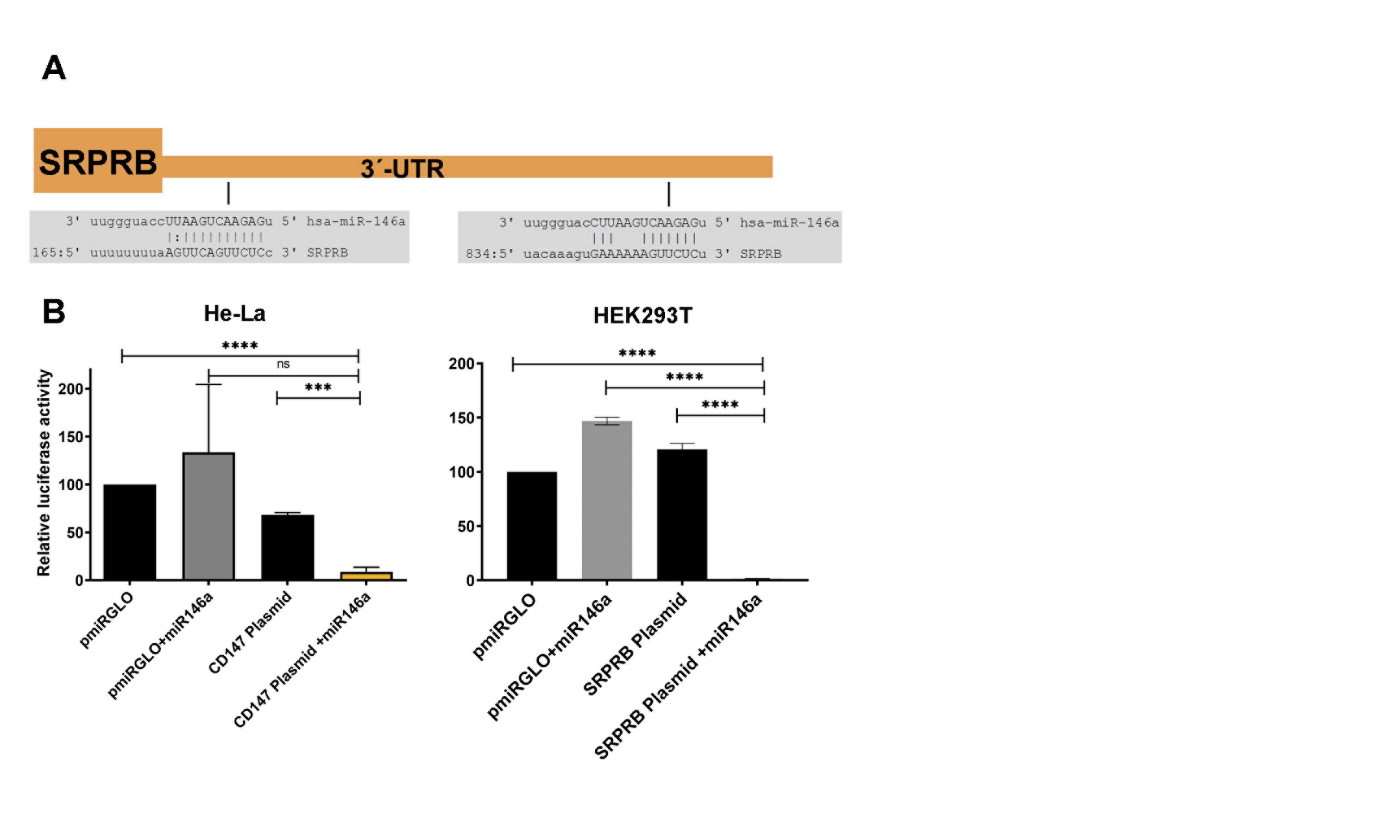
**

**Supplemental Figure 5**. Luciferase reporter assays for SRPRB as direct miR-146a target gene**. A.** Diagram illustrates miR-146a binding domains within the 3´-UTR of *SRPRB* gene, binding sites were calculated using miRNA target prediction tool miRanda. **B.** HEK293T cells were transfected with pmirGLO Dual-Luciferase miRNA target expression vector with and without *SRPRB* binding site and additionally with and without miR-146a mimic. After transfected for 40 h, luciferase activity was measured in triplicates in two independent experiments by a dual-luciferase reporter assay (firefly LUC / renilla LUC). Columns represent mean luciferase activity values of two experiments in triplicates of HEK293T cells transfected with a vector containing the CD147 or SRPRB 3´-UTR fraction overexpressing miR-146a relative to not overexpressing miR-146a cells. Unpaired t-test, ***p<0.001 ****p<0.0001.


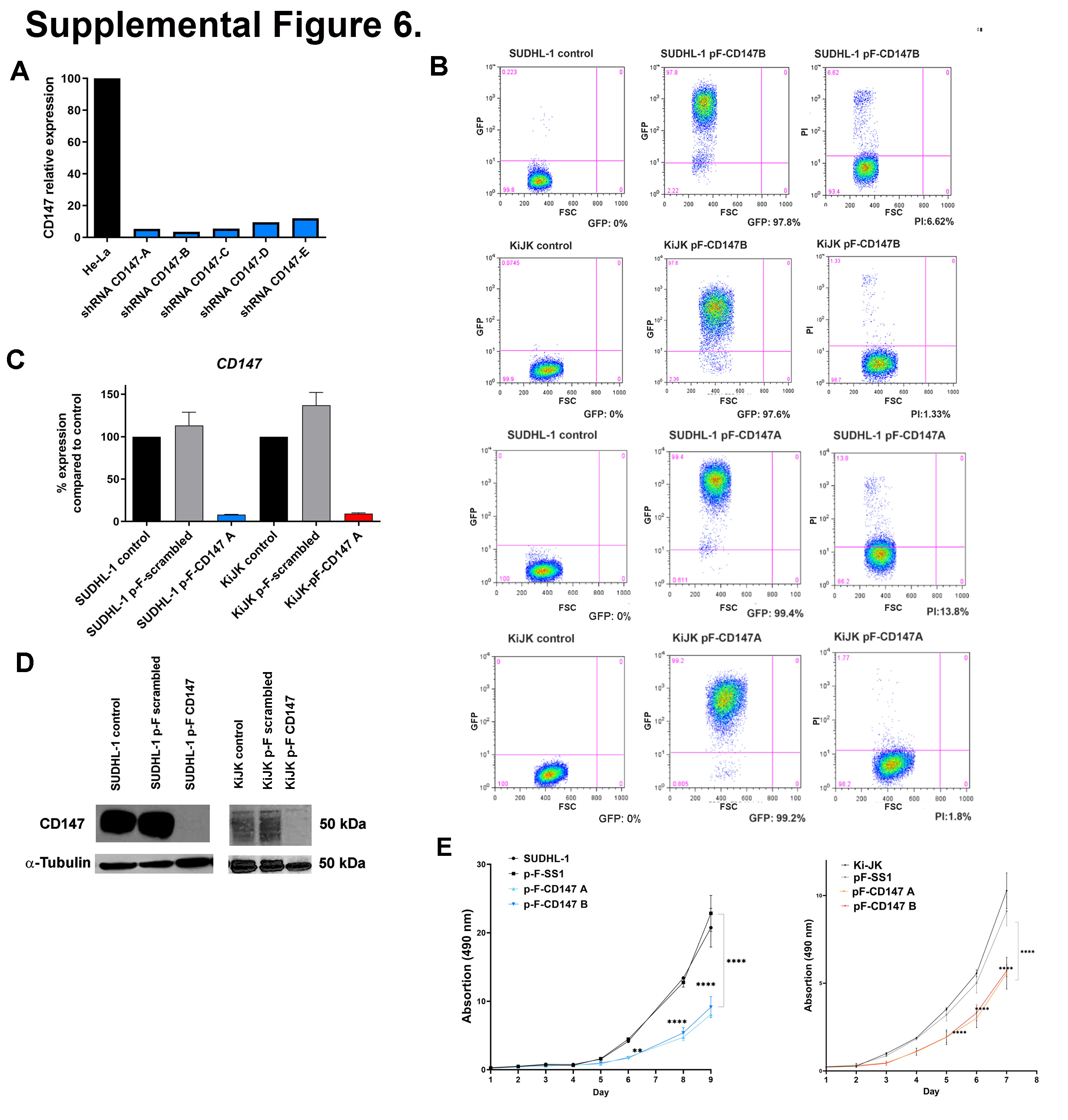


**Supplemental Figure 6.** Analysis of the efficiency of CD147 shRNA and validation of its knockdown in ALCL cells. **A.** Endogenous CD147 knockdown in HeLa cells using different CD147 shRNAs. RT-qPCR analysis of CD147 expression. mRNA values were normalized to ACTB and data were analyzed according to the 2^− Δ Δ Cp^ method. Results are depicted as mRNA amount relative to untreated HeLa cells**. B.** Flow cytometric analysis of transduced SUDHL-1 and KiJK cells and untreated controls three days after second infection. The percentage of GFP-positive cells represents the percentage of infected cells whereas the percentage of cells positive for propidium iodide (PI) indicate the dead cells. **C**. RT-qPCR analysis of CD147 mRNA in the transduced SUDHL-1 and KiJK cells three days after second infection. Values were normalized to ACTB and data were calculated according to the 2^−ΔΔCp^ method. Results are represented as mRNA amount relative to untreated cells. **D.** Western Blot analysis of CD147 in SUDHL-1 and KiJK cells transduced with CD147 shRNA four days after infection. Thirty µg protein were loaded to detect CD147. α-tubulin was used as loading control. These blots are representative of the biological triplicates **E.** Proliferation curves of the controls and CD147-shRNA infected SUDHL-1 and KiJK cells are depicted up to 8 days after infection. Error bars indicate SEM (n = 3). SUDHL-1 control= uninfected cells, pF -scrambled=virus containing non-targeted shRNA sequence, pF-CD147 A= virus containing the CD147 shRNA sequence A. Statistical analysis was performed using unpaired t-test (for only two group comparison) and repeated measures ANOVA (pFCD147 vs pF, pF-S, and pFSS1) significant differences at different time points and ending point are indicated as **, P<0.001 ****, *P*<0.0001.


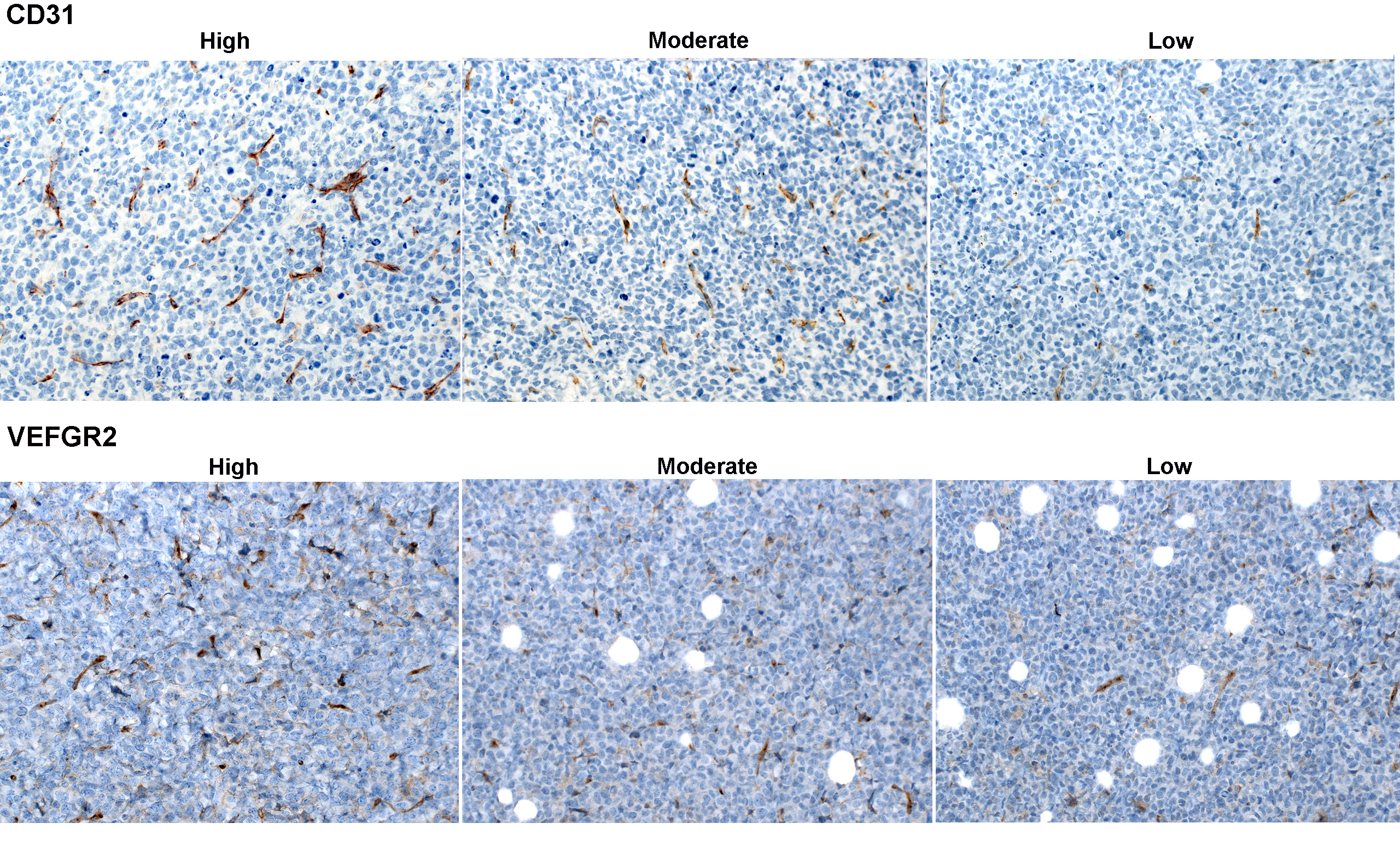
**Supplemental Figure 7.** CD31 and VEFGR2 immunohistochemistry scale for quantification. Tumor areas were assessed according the following criteria. High: areas > 20 visible large vessels or >30 small vessels; Moderate: areas with 10 to 30 small or large vessels; low: areas with less than 15 small vessels (CD31 and VEFGR2, Immunohistochemistry, original magnification 200x).


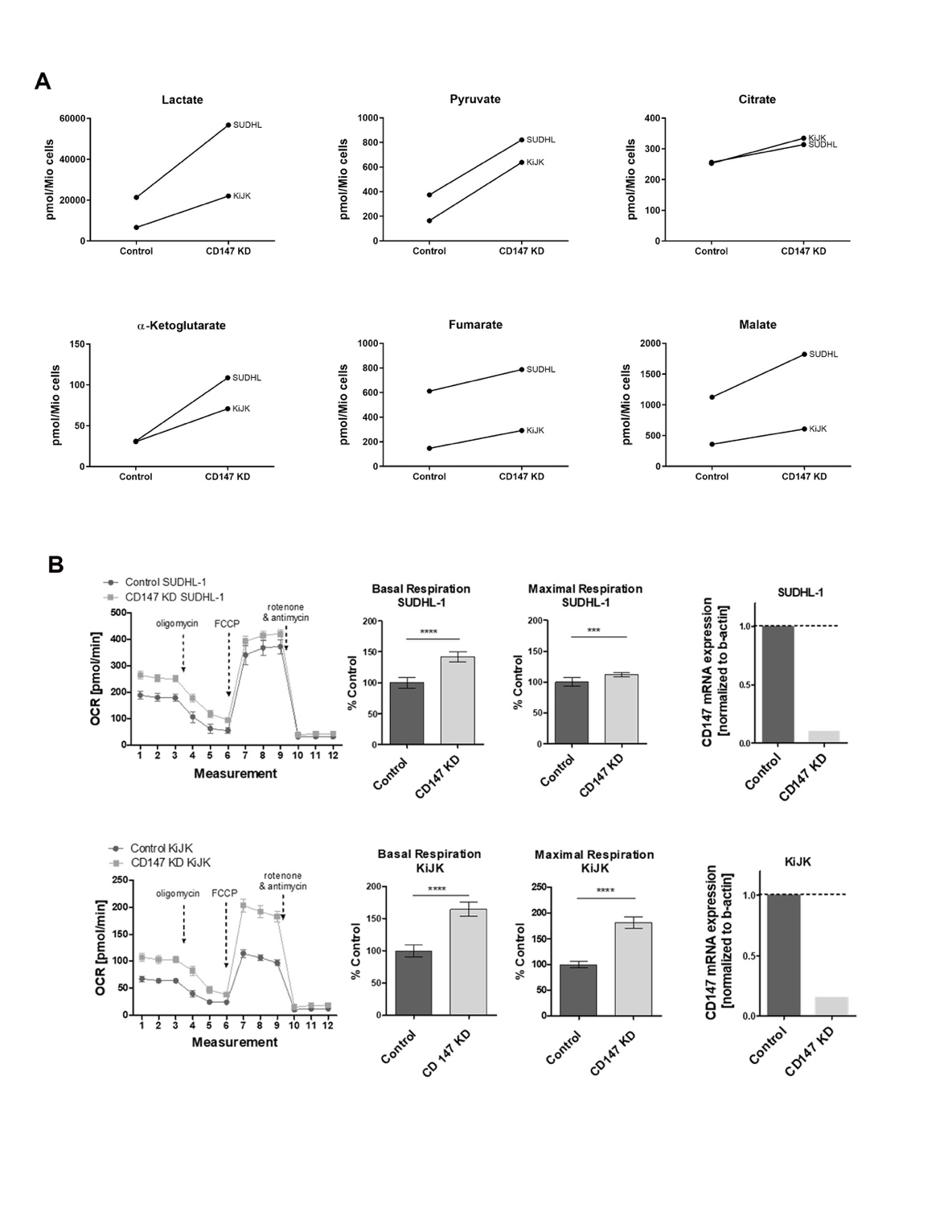


**Supplemental Figure 8.** Investigation of metabolic alterations upon CD147 knockdown *in vitro*. **A**. Intracellular concentration of lactate and TCA cycle intermediates in SUDHL-1 and KiJK CD147-KD cells compared to controls (n=1 biological replicate per cell line). **B.** Altered oxygen consumption rate in SUDHL-1 and KiJK KD cells compared to controls (n=1 biological replicate per cell line). CD147 knockdown was verified through qPCR in cells used for analyses of metabolic alterations. Unpaired t-test, *p<0.05, **p<0.01, ***p<0.001, ****p< 0.0001.

**References**

1. Drexler HG, MacLeod RA. Malignant hematopoietic cell lines: in vitro models for the study of anaplastic large-cell lymphoma. *Leukemia* 2004 Oct; **18**(10)**:** 1569-1571.

2. Koch I, Slotta-Huspenina J, Hollweck R, Anastasov N, Hofler H, Quintanilla-Martinez L*, et al.* Real-time quantitative RT-PCR shows variable, assay-dependent sensitivity to formalin fixation: implications for direct comparison of transcript levels in paraffin-embedded tissues. *Diagn Mol Pathol* 2006 Sep; **15**(3)**:** 149-156.

3. Dejean E, Renalier MH, Foisseau M, Agirre X, Joseph N, de Paiva GR*, et al.* Hypoxia-microRNA-16 downregulation induces VEGF expression in anaplastic lymphoma kinase (ALK)-positive anaplastic large-cell lymphomas. *Leukemia* 2011 Dec; **25**(12)**:** 1882-1890.

4. Anastasov N, Bonzheim I, Rudelius M, Klier M, Dau T, Angermeier D*, et al.* C/EBPbeta expression in ALK-positive anaplastic large cell lymphomas is required for cell proliferation and is induced by the STAT3 signaling pathway. *Haematologica* 2010 May; **95**(5)**:** 760-767.

5. Biegler B, Kasinrerk W. Reduction of CD147 surface expression on primary T cells leads to enhanced cell proliferation. *Asian Pac J Allergy Immunol* 2012 Dec; **30**(4)**:** 259-267.

6. Chen X, Lin J, Kanekura T, Su J, Lin W, Xie H*, et al.* A small interfering CD147-targeting RNA inhibited the proliferation, invasiveness, and metastatic activity of malignant melanoma. *Cancer Res* 2006 Dec 01; **66**(23)**:** 11323-11330.

7. Anastasov N, Klier M, Koch I, Angermeier D, Hofler H, Fend F*, et al.* Efficient shRNA delivery into B and T lymphoma cells using lentiviral vector-mediated transfer. *J Hematop* 2009 Mar; **2**(1)**:** 9-19.

8. Sanjana NE, Shalem O, Zhang F. Improved vectors and genome-wide libraries for CRISPR screening. *Nature methods* 2014; **11**(8)**:** 783-784.

9. Robinson JT, Thorvaldsdóttir H, Winckler W, Guttman M, Lander ES, Getz G*, et al.* Integrative genomics viewer. *Nature Biotechnology* 2011 01/10/online; **29:** 24.

10. Wolburg-Buchholz K, Mack AF, Steiner E, Pfeiffer F, Engelhardt B, Wolburg H. Loss of astrocyte polarity marks blood-brain barrier impairment during experimental autoimmune encephalomyelitis. *Acta Neuropathol* 2009 Aug; **118**(2)**:** 219-233.

11. Yu YR, Imrichova H, Wang H, Chao T, Xiao Z, Gao M*, et al.* Disturbed mitochondrial dynamics in CD8(+) TILs reinforce T cell exhaustion. *Nat Immunol* 2020 Dec; **21**(12)**:** 1540-1551.
